# Generation of 12-Lead Electrocardiogram Using Smartwatches in Patients With Cardiac Conditions: Clinical Validation Study

**DOI:** 10.2196/86621

**Published:** 2026-11-05

**Authors:** Mauro Buelga Suárez, Carmen Plaza-Seco, Roberto Matía Francés, Gonzalo Luis Alonso Salinas, Manuel Blanco-Velasco

**Affiliations:** 1 Cardiology Department, Hospital Universitario Ramón y Cajal, Madrid, Spain; 2 Medicine and Health Sciences Department, University of Alcalá, Alcalá de Henares, Madrid, Spain; 3 Ramón y Cajal Institute for Health Research (IRYCIS), Madrid, Spain; 4 Department of Signal Theory and Communications, Universidad de Alcalá, Alcalá de Henares, Madrid, Spain; 5 Department of Cardiology, Faculty of Health Sciences, Universidad Publica de Navarra, Irunlarrea 3, Pamplona, 31008, Spain, +34 848429503; 6 Health Sciences Department, Universidad Pública de Navarra, Pamplona, Spain; 7 Navarrabiomed (Miguel Servet Foundation), Instituto de Investigación Sanitaria de Navarra (IdiSNA), Pamplona, Spain

**Keywords:** electrocardiography, ambulatory monitoring, telediagnostics, augmented leads, smartwatch

## Abstract

**Background:**

Smartwatches have significantly expanded personal health monitoring capabilities; however, their application in comprehensive cardiac assessment remains limited by single-lead electrocardiogram (ECG) acquisition. The inability to directly obtain augmented limb leads represents a major limitation for the evaluation of ischemic and arrhythmogenic conditions.

**Objective:**

This study aimed to present and preliminarily evaluate a methodology for generating augmented limb leads (augmented voltage right [aVR], augmented voltage left [aVL], and augmented voltage foot [aVF]) from sequential smartwatch recordings through signal synchronization and RR interval alignment, and to combine this approach with precordial smartwatch recordings to reconstruct a full 12-lead ECG in a proof-of-concept setting.

**Methods:**

A signal-processing framework was developed to preserve ECG morphology across sequential smartwatch lead recordings. The method is based on RR interval–guided temporal resampling, enabling interlead synchronization and analytical reconstruction of augmented limb leads. A prospective proof-of-concept clinical validation was conducted in 20 cases with diverse cardiac pathologies, including 1 healthy control. Smartwatch-derived ECGs were compared with standard 12-lead ECGs. Evaluation included expert-based clinical interpretation by 85 health care professionals and objective quantitative analysis of key electrocardiographic parameters, including heart rate, PR interval, QRS duration, QT interval, QTc, RR interval, P-wave duration, and PP interval. Mean values, SDs, and intermethod differences were calculated to assess agreement and variability.

**Results:**

Reconstruction of a 12-lead ECG from smartwatch-acquired signals was successfully achieved in all cases. Visual correlation scores between smartwatch-derived and conventional ECGs ranged from 78.1% to 91.3% across specialty-level means (mean 81.1, SD 22.7), indicating high perceived concordance; individual case-level scores ranged more widely, from 47.0% to 96.3%. Evaluator-perceived axis agreement reached 95.4% across all cases, while the investigators’ direct axis measurements showed complete concordance in all cases. Quantitative analysis demonstrated small mean differences between modalities, with particularly high agreement observed in structurally stable parameters such as heart rate (mean 77.2, SD 22.3 vs mean 75.9, SD 23.1 bpm; Δ=1.3 bpm) and PR interval (mean 176.2, SD 29.1 vs mean 180.8, SD 19.8 ms; Δ=−4.6 ms). Greater variability was observed in atrial activation parameters such as P-wave duration (Δ=−13.3 ms) and time-dependent parameters such as QT (Δ=−19.2 ms) and QTc (Δ=−12.7 ms).

**Conclusions:**

This proof-of-concept study demonstrates the feasibility of reconstructing a full 12-lead ECG from smartwatch-derived signals using a synchronization-based approach. The results demonstrated generally small mean differences between conventional and smartwatch-derived ECG measurements, although larger interval differences were observed in some individual cases. Importantly, due to the sequential nature of signal acquisition, the proposed methodology does not aim to achieve direct waveform equivalence but rather clinically meaningful agreement in electrocardiographic parameters. These findings support the potential of smartwatches as accessible tools for advanced cardiac monitoring and broaden their applicability beyond single-lead screening.

## Introduction

In recent decades, rapid advances in technology have enabled the integration of advanced biometric sensors into everyday devices, such as smartwatches. Among the most notable innovations is the incorporation of electrocardiogram (ECG) sensors [1] capable of monitoring the electrical activity of the heart in real time.

Previously limited to functions such as monitoring heart rate and recording physical activity, these devices now offer an accessible tool for detecting potentially serious cardiac abnormalities such as atrial fibrillation (AF) [2-5]. Similarly, some studies have shown that variations in ECG patterns recorded by smartwatches, although limited in their diagnostic capability compared with high-precision medical equipment, may be useful in alerting users to possible cardiac abnormalities. Some devices have detected subtle changes in ST-segment or T-wave irregularities, which, in medical contexts, are considered classic indicators of ischemia and may help identify early ischemic episodes [6,7] and contribute to the detection of ventricular tachycardia (VT) [8,9].

Smartwatches are equipped with specialized software and algorithms that generate electrocardiographic waveforms comparable to those of a single lead (lead I) in a traditional ECG. By applying the basic principles of electrocardiography to smartwatches, it is possible to obtain 9 ECG leads. In the frontal plane, with these devices, only the limb leads (I, II, and III) can be obtained directly. The augmented leads (augmented voltage right [aVR], augmented voltage foot [aVF], and augmented voltage left [aVL]) cannot be calculated directly due to the inability to generate the central terminal (Wilson Central Terminal [WCT]) [10]. This inability to directly derive augmented leads poses a significant diagnostic limitation for cardiac pathologies of arrhythmogenic and ischemic origin, particularly in cases such as inferior infarction (II, III, aVF) or those with a high-lateral location (I, aVL). The main objective of this study is to present and preliminarily evaluate the use of smartwatches for obtaining 12-lead ECGs by generating augmented leads (aVR, aVL, and aVF) from sequentially recorded smartwatch signals. This process uses advanced techniques, including R peak alignment, interpolation, filtering, and signal synchronization. Unlike previous work that averaged beats into a single representative complex [11,12], that compared sequential smartwatch leads without interlead synchronization [13], or that reconstructed missing leads using learning-based generative models [14], our approach uniquely preserves beat-level morphology by aligning RR intervals across sequential recordings and analytically deriving the augmented leads. As secondary objectives, the following were established:

1. Integration of this technique aligns the frontal plane leads with the horizontal plane leads (V1-V6), enabling the reconstruction of a fully synchronized 12-lead ECG.
2. Assessment of the performance in 20 real cases. This methodological validation focused on signal acquisition and processing rather than patient outcomes, with a single institution deliberately chosen to ensure homogeneous conditions and procedural control.

A European patent for this work has been filed under reference EP25382712.5.

## Methods

### Data Acquisition

In a standard ECG, 12 different leads are recorded simultaneously, each of which provides a detailed view of the electrical activity of the heart from various angles. This simultaneous approach allows for a comprehensive analysis of cardiac electrical behavior. There are 6 leads in the frontal plane, consisting of 3 limb leads (I, II, and III) and 3 augmented limb leads (aVR, aVF, and aVL). The limb leads are the original leads chosen by Einthoven in 1901 [15] to record electrical potentials in the frontal plane. Electrodes are placed on the left arm, right arm, and left leg. These leads are obtained from the difference in electrical potential between each electrode placed on one of the three extremities and the WCT, which is essentially the barycenter of Einthoven’s equilateral triangle with a potential very close to infinity (zero). Limb leads are bipolar because they represent an electrical potential difference between 2 poles, positive and negative, with the lead axis being the line connecting the 2 poles (each line is divided into 2 halves, one positive half near the positive pole and one negative half near the negative one). The 3 limb leads are:

- Lead I: potential difference between the left arm (LA) and right arm (RA) (LA − RA).
- Lead II: potential difference between the left leg (LL) and right arm (LL − RA).
- Lead III: potential difference between the left leg and the left arm (LL − LA).

In the horizontal plane, there are 6 leads, called precordial leads (V1, V2, V3, V4, V5, and V6), that measure the electrical activity of the heart at right angles to the frontal plane. They are obtained by placing the electrodes in preset areas of the anterior chest wall. A precordial lead measures the electrical potential difference between a thoracic electrode and the WCT.

When these principles of electrocardiography are used as the basis for a smartwatch, only the limb leads (I, II, and III) can be obtained directly. The augmented leads (aVR, aVF, and aVL) cannot be calculated directly because of the inability to generate the WCT. The polarity of the leads is determined by electrode placement on smartwatches [16].

To obtain them, the following approach, shown in Figure 1, is used:

1. The ECG recording of the smartwatch is obtained using its integrated electrodes. The digital crown (negative) and the back crystal (positive) serve as electrodes.
2. To obtain lead I, the watch is placed on the left wrist (back crystal, positive electrode), and the right index finger touches the digital crown (negative electrode).
3. Leads II and III are obtained with the back of the watch (back crystal, positive electrode) against the lower-left abdomen and the right index finger (lead II) or left index finger (lead III) touching the crown (negative electrode).
4. To obtain precordial leads with the smartwatch, the back of the watch (back crystal, positive electrode) is placed in the classic positions for the precordial leads V1-V6, with the right index finger touching the crown (negative electrode).

**Figure 1.**
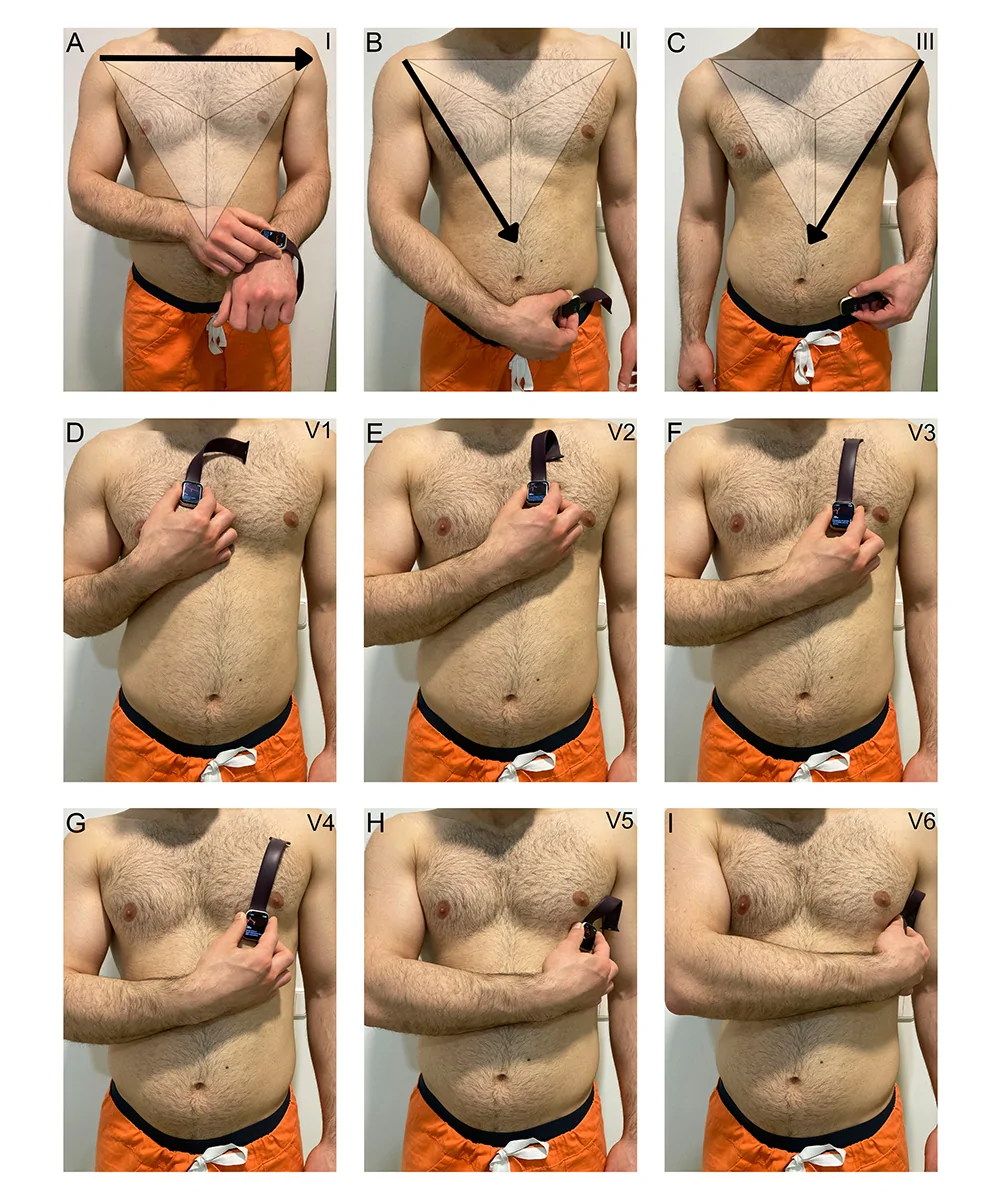
Schematic of smartwatch placement for obtaining leads I, II, and III (A-C) in the frontal plane and V1 to V6 (D-I) in the horizontal plane. Lead I: potential difference between the left arm and right arm (LA − RA). Lead II: potential difference between the left leg and right arm (LL − RA). Lead III: potential difference between the left leg and the left arm (LL − LA). LA: left arm; LL: left leg; RA: right arm.

Similar to the augmented leads, the absence of the WCT should theoretically prevent the recording of precordial leads. In this case, smartwatches create a closed positive/negative circuit at positions V1 to V6 [17], allowing the recording of each lead [5]. However, it should be noted that this configuration does not reproduce a true unipolar precordial lead referenced to the WCT, but rather generates a bipolar recording, which represents an approximation of conventional precordial leads [13].

Unlike conventional electrocardiographs, smartwatches cannot record the electrical activity of the heart simultaneously. With these devices, the leads are obtained sequentially, and to calculate the augmented leads, it is necessary to have previously recorded the limb leads (I, II, and III). By recording the leads sequentially, 3 independent electrocardiographic tracings are obtained, each with a slightly different heart rate.

Additionally, a representative case of Brugada syndrome recorded during a flecainide test was included as an illustrative example. This case was retrospectively selected from routine clinical practice and was not part of a predefined study protocol or a systematic pharmacological intervention.

### ECG Signal Alignment and Augmented Lead Generation

Leads I, II, and III, recorded with the smartwatch, served as the basis for this analysis. From these, the augmented leads aVR, aVL, and aVF were calculated, considering only the frontal plane. The smartwatch provides sequential 30-second recordings, whereas conventional electrocardiographs generate 10-second segments. To ensure standardized comparison, the first 10 seconds of each lead obtained with the smartwatch were used. Subsequently, for the reconstruction of the 12-lead ECG, the signals were organized in a 3 × 4 format [18], following the conventional procedure in electrocardiography. In this format, 10 seconds of continuous recording are represented, divided into columns of 2.5 seconds: the first column starts at 0 seconds, the second at 2.5 seconds, the third at 5 seconds, and the fourth at 7.5 seconds. Thus, the electrical activity of all leads is displayed over a continuous 10-second interval, facilitating analysis and comparison with recordings obtained using standard electrocardiographs.

The first step involves detecting the QRS complexes and R peaks in each smartwatch lead using an AI-based detector developed by our group and previously validated [19]. All detections are manually reviewed in challenging cases to ensure accuracy.

In this study, lead I serves as the reference, with its R peak positions guiding the alignment of the other leads. We use the heart rate variability (HRV), which quantifies the temporal fluctuations between successive R peaks, to evaluate differences between leads. Thus, HRV is obtained by interpolating the interval function *d*(*t*), which is considered to be a nonuniformly sampled signal of the variability function [20]:


(1) $$d\left(t\right)\text{=}\;\sum_{k\text{=}1}^{B}\left(t_{k}-t_{k-1}\right)\delta \left(t-t_{k}\right)$$

Here, *t*_*k*_ represents the time of the *k*th detected R peak, δ(*t*) is the Dirac delta function that indicates the location of each beat, and *B* is the number of beats in the sequence. Ideally, the HRV curves for all 3 leads would be the same, which is not the case due to sequential recording, as can be seen in the bottom-left graph of Figure 2.

**Figure 2.**
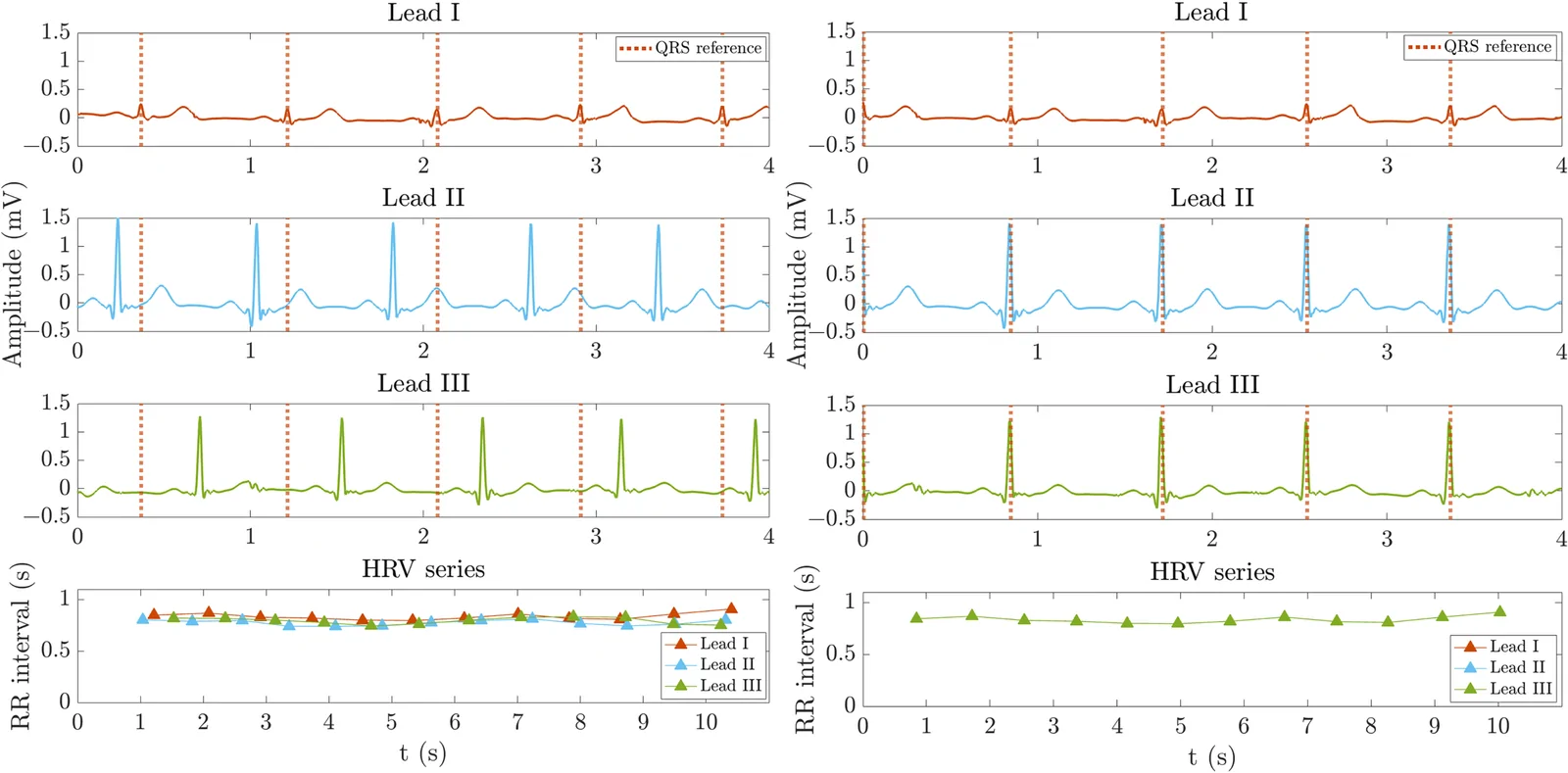
ECG smartwatch leads I, II, and III (4 s out of the total 10 s window) and the corresponding HRV for the entire 10-second segment from a healthy control case. The left plot shows misaligned leads with nonoverlapping HRV curves. The right plot shows the aligned leads after the resampling process, with all HRV curves overlaid and aligned with each other. ECG: electrocardiogram; HRV: heart rate variability.

To align leads II and III with lead I, we resample the RR intervals to make them equal, ensuring that interbeat distances match those of lead I. This process is carried out by applying a sampling frequency change scheme to adjust the number of samples in each RR block and make them equal to the reference lead; it may be seen as an RR normalization procedure. Given that the resampling process is implemented for each RR block separately, we consider $x_{l}^{\left(s\right)}[n]$, *n*=0, 1, ..., *L*_*s*_− 1, as the *s*th RR block of lead *l*, where *l* ∈ {I, II, III}, so the ECG signal for lead *l* can be expressed as the concatenation of successive RR blocks:


(2) $$x_{l}\left[n\right]\text{=}\;x_{l}^{\left(0\right)}\left[n\right]\text{+}\;\sum_{s\text{=}1}^{K-1}x_{l}^{\left(s\right)}\left[n-\sum_{i\text{=}0}^{s-1}L_{i}\right]$$

where *K*(*B −* 1) is the total number of RR intervals, and *L_i_* is the length of the *i*th segment.

The resampling process, illustrated in Figure 3, involves three key steps [21]. First, each segment $x_{l}^{\left(s\right)}[n]$ (for *l*=II, III) is upsampled by a factor *L*, increasing the number of samples. Next, a low-pass filter is applied to the upsampled segments to remove high-frequency artifacts introduced during interpolation, ensuring the signal remains within the Nyquist limit. Finally, the filtered segments are downsampled by a factor M to match the number of samples in lead I.

**Figure 3.**
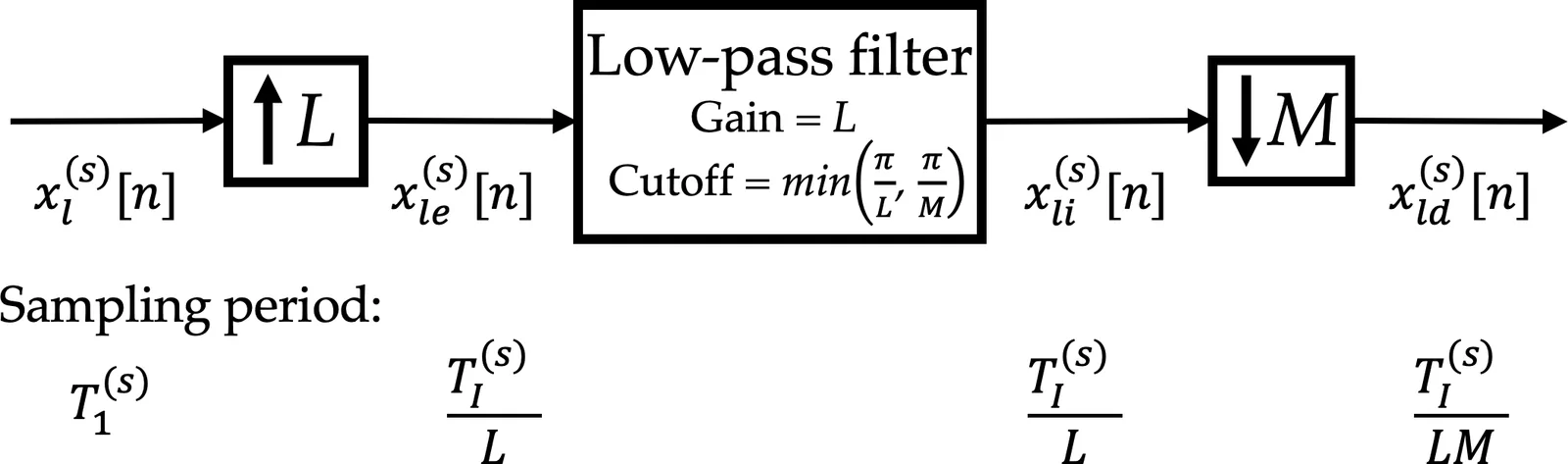
System for resampling by a noninteger factor, illustrating the combined processes of interpolation, filtering, and decimation.

This combination of interpolation, filtering, and decimation adjusts the sampling rate of each segment by a noninteger factor, ensuring that the RR intervals of leads II and III align with those of lead I. Importantly, the overall sampling frequency of the signal remains unchanged. By resampling each RR segment individually, this method preserves the temporal and morphological details of the ECG signal, ensuring that the QRS complexes and R peaks in leads II and III align with those in lead I. Since the ECG is oversampled, the waveform does not change visually. Thus, the amplitudes of the P, R, and T peaks remain the same, and the time scale is adjusted proportionally; that is, the scale is preserved across the entire interval. Unlike averaging techniques [11,12], which may mask pathological features, this approach minimizes information loss and maintains their integrity. Recent studies [13] have shown that smartwatch recordings obtained at various limb and precordial positions can yield amplitude and duration measurements comparable to standard 12-lead ECGs, but those signals are still acquired sequentially and compared lead-by-lead without alignment or RR-block resampling to synchronize morphology across leads. Similarly, Chen et al [14] propose a learning-based model that reconstructs a full 12-lead ECG from a single lead, generating new information to approximate the missing signals. In contrast, our method operates directly on the patient’s recorded signals. RR-block resampling ensures interlead temporal consistency and preserves beat-level morphology, enabling the subsequent derivation of augmented leads. Once the primary leads are aligned, the augmented leads are calculated using Goldberger’s equations [22]:


(3) $$\text{aVR}=-\frac{\text{I+II}}{2}$$

(4) $$\text{aVL}=\frac{\text{I}-\text{III}}{2}$$

(5) $$\text{aVF}=\frac{\text{II+III}}{2}$$

This alignment ensures that each augmented lead is synchronized with the primary ECG signals, improving the reliability of subsequent analyses and enabling direct comparison with conventional 12-lead ECGs. The signal-processing script used to generate the augmented limb leads has been provided as Multimedia Appendix 1.

### Obtaining 12-Lead ECG

The alignment process described for leads II and III can be generalized to include the precordial leads (V1-V6), enabling the reconstruction of a full 12-lead ECG. Using lead I as the reference, the same resampling methodology can be applied to align the precordial leads. This involves transforming the nonsynchronized RR intervals from leads V1 to V6 into synchronized representations, ensuring their cardiac intervals match those of lead I.

Let the *s*th block from leads V1-V6 be denoted by $x_{v_{k}}^{\left(s\right)}[n]$, where *v*_*k*_ ∈ {V1, V2, V3, V4, V5, V6}. Each RR interval has a different period, $T_{v_{k}}^{\left(s\right)}$, and the complete signals are defined as the concatenation of all segments (Equation 2). The same resampling process is applied to align the precordial leads with lead I. By aligning all leads (I, II, III, and V1-V6) obtained from the smartwatch, along with the generated augmented leads, the smartwatch ECG signals can be directly compared with conventional 12-lead ECG recordings. This generalization of the alignment process ensures that the precordial leads are synchronized with the primary leads, enabling a comprehensive analysis of the full 12-lead ECG. Such alignment improves the reliability of diagnostic comparisons between smartwatch-derived ECGs and standard clinical ECGs.

### Clinical Validation

A prospective observational and comparative proof-of-concept study was conducted to validate augmented leads (aVR, aVL, and aVF) derived from Apple Watch Series 9 recordings against those from a conventional electrocardiograph. Twenty nonconsecutive cases were included. Of these, 19 patients admitted to cardiology presented diverse cardiac pathologies and evident pathological electrocardiographic alterations in the frontal plane. In addition, one healthy control in sinus rhythm without known cardiac disease was included. The 12-lead ECGs were obtained using the previously described process.

It should be noted that the expert-based clinical validation in this study was limited to frontal plane leads, as these represent the primary focus of the proposed methodological contribution, namely the analytical reconstruction of augmented leads.

To complement this expert-based evaluation, a quantitative analysis was performed comparing conventional ECG (A) and smartwatch-derived ECG (B) across all cases. Standard electrocardiographic parameters were evaluated, including heart rate, PR interval, QRS duration, QT interval, QTc, RR interval, P-wave duration, and PP interval. All measurements were performed by experienced cardiology researchers using digital ECG analysis tools, ensuring consistent interval and waveform assessment across both modalities. For each parameter, mean values and SDs were calculated for both modalities across all cases, along with the mean difference between methods (A − B). This approach allowed the assessment of both central tendency and dispersion, providing an objective evaluation of agreement and variability between conventional and smartwatch-derived ECG recordings.

Additionally, a questionnaire was anonymously distributed to 85 health care professionals experienced in electrocardiographic diagnosis, comprising 50 cardiologists, 5 internists, 10 family physicians, 5 emergency physicians, and 15 critical care nurses. This comparative approach used data from both the Apple Watch Series 9 and a Philips TC30 conventional electrocardiograph (Koninklijke Philips N.V.) to assess the concordance and clinical utility of the smartwatch-generated augmented leads.

### Questionnaire

To collect expert clinical opinions on the agreement and diagnostic concordance of electrocardiographic signals, a structured questionnaire was used. For each of the 20 cases, whose diagnoses were confirmed and supported by a clinical electrophysiologist, an image was presented with two ECG options, including only the frontal plane leads. These options, labeled “Option A” and “Option B,” randomly corresponded to either the conventional electrocardiograph recording or the smartwatch-generated tracing. The questionnaire comprised 5 key questions (Figure S1 in Multimedia Appendix 1) designed to assess specific aspects related to method agreement and experts’ perception of diagnostic concordance:

1. Diagnosis of electrocardiographic tracings: Evaluators selected from 20 available diagnostic options, acknowledging that some tracings might present multiple diagnoses due to coexisting alterations. This assessment included tracings from both smartwatches and conventional electrocardiographs.
2. Determination of the electrical axis of the cardiac: Evaluation and comparison of the electrical axis calculated using both technologies, based on the polarity of the QRS complex in leads I and aVF.
3. Diagnostic concordance between both technologies: Assessment of the level of agreement in diagnoses between smartwatch-derived ECGs and conventional ECG systems.
4. Visual correlation assessment of the tracings: Evaluators assigned a percentage score (1%-100%) to indicate the perceived degree of visual correlation between tracings obtained from both technologies. It should be noted that this “correlation score” represents a subjective visual assessment of morphological similarity and does not correspond to a mathematically computed correlation coefficient.
5. Identification of the tracing source (smartwatch or electrocardiograph): Assessment of clinicians’ ability to correctly distinguish between tracings obtained from a smartwatch and those from a traditional electrocardiograph.

The results were included in the centralized database for subsequent analysis.

### Ethical Considerations

This study was conducted in accordance with the ethical principles outlined in the Declaration of Helsinki (World Medical Association, 2013) and its subsequent amendments. The study protocol was reviewed and approved by the Ethics Committee of Ramón y Cajal University Hospital (Madrid, Spain) on July 31, 2023 (approval number 181/23). All participants provided written informed consent prior to enrollment. Patient data were fully anonymized, and all study data were securely stored in compliance with applicable data protection regulations. No information that could reveal the identity of any participant has been included in this manuscript.

## Results

Data supporting the findings of this study are provided in Multimedia Appendix 1.

### 12-Lead ECG

To demonstrate an example of the methodology used to align the leads and generate augmented leads, we present a case of type I Brugada syndrome. Brugada syndrome is a cardiac pathology characterized by specific ECG patterns, including ST-segment elevation and T-wave inversions, and is associated with an increased risk of sudden cardiac death. This pathology provides an interesting scenario for comparing the 12-lead smartwatch-derived ECG with the conventional 12-lead ECG.

Figure 4 shows the results. Figure 4A shows the conventional 12-lead ECG recorded simultaneously from the same patient. Figure 4B displays the 12-lead ECG recorded with the smartwatch after alignment and generation of augmented leads, following the described methodology.

**Figure 4.**
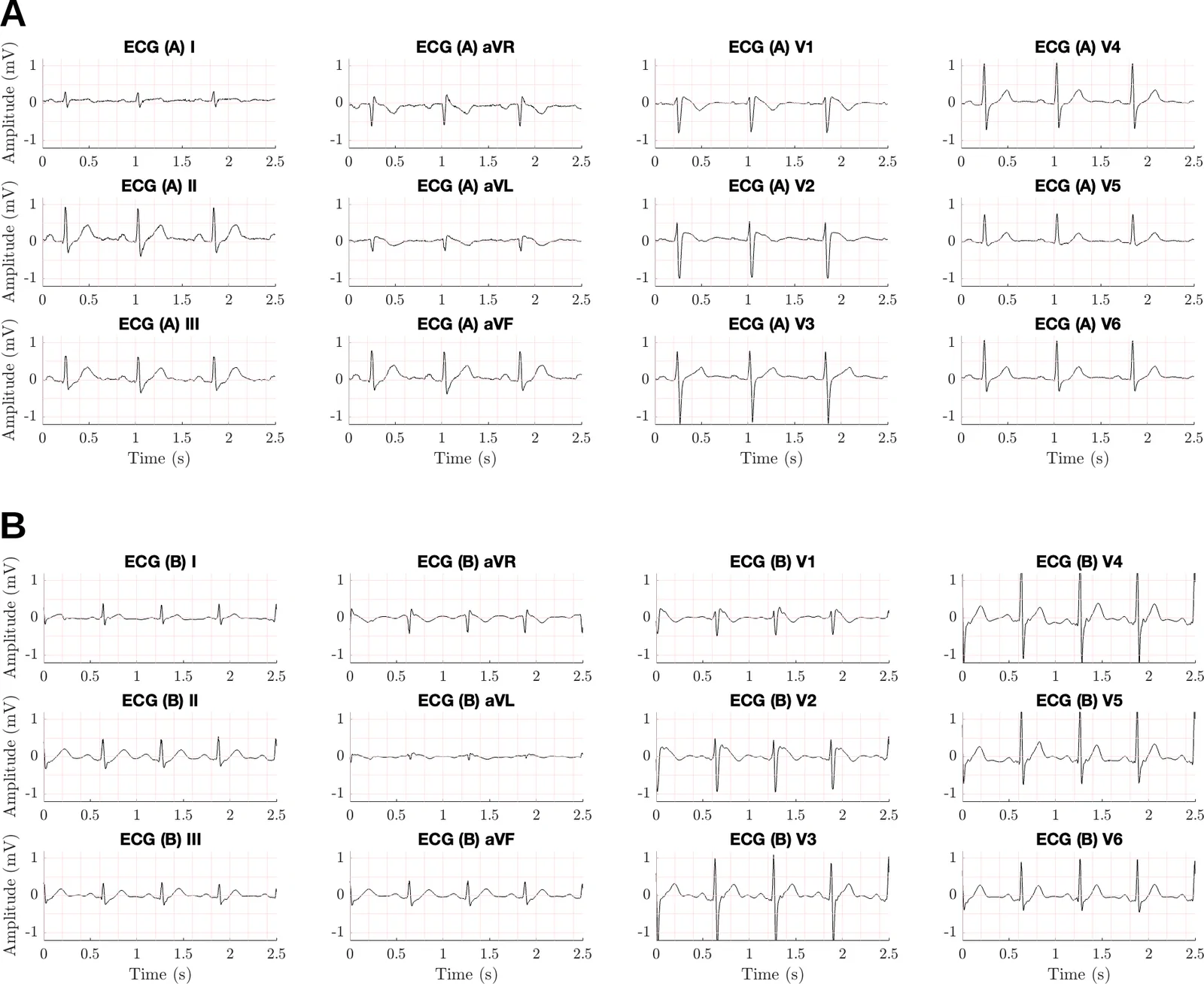
Brugada type I pattern during flecainide test. Conventional 12-lead ECG (A) and smartwatch 12-lead ECG (B). ECG: electrocardiogram.

Both ECGs are highly similar, demonstrating the accuracy of the reconstruction process. In both recordings, the characteristic Brugada type I pattern is clearly preserved, with typical ST-segment elevation in the right precordial leads (V1-V3). In addition, the corresponding morphology of the augmented limb leads (aVR, aVL, and aVF) is maintained, supporting the overall consistency of the reconstructed 12-lead ECG.

### Clinical Validation

Each of the 20 cases underwent an independent review by an expert arrhythmologist. Detailed case-by-case clinical interpretations and representative figures are provided in Multimedia Appendix 1.

The analysis of data from the 20 cases studied shows variable outcomes depending on the specialties of the evaluators and the complexity of the cases. In general, diagnostic accuracy, axis concordance, and the consistency between diagnoses obtained from conventional leads and those recorded by the experimental device are consistent across specialties, although significant differences have been observed in certain cases.

The average perceived diagnostic concordance is 79.4%. Emergency specialists showed the highest agreement (90%), followed by cardiologists (80.2%) and internal medicine specialists (80.0%). General practitioners and nurses showed lower levels of agreement at 77.5% and 74.3%, respectively. Complex cases, such as cases 9 and 10, demonstrate lower general concordance, particularly in noncardiology specialties, reflecting the inherent difficulty in interpreting certain patterns.

The average evaluator-perceived concordance along the axis between diagnoses performed with the experimental device and the standard method is 95.4%, distinct from the investigators’ direct axis measurements, which showed complete concordance in all cases. Emergency specialists show the highest concordance (99%), followed by cardiologists (98.1%) and general practitioners (96.0%). Although nurses and internal medicine specialists show slightly lower values (86.7% and 90%, respectively), they maintain high general concordance. The correlation scores range from 78.1 to 91.3 across specialty-level means, with an overall mean of 81.1 (SD 22.7). Emergency specialists show the highest scores (mean 91.3, SD 24.2), followed by internal medicine specialists (mean 84.2, SD 23.4) and cardiologists (mean 81.0, SD 22.2). Nurses and general practitioners obtain mean lower scores of 79.0 (SD 21.2) and 78.1 (SD 24.8), respectively (Figure 5). At the individual case level, scores ranged more widely, from 47.0 (case 10) to 96.3 (case 2).

**Figure 5.**
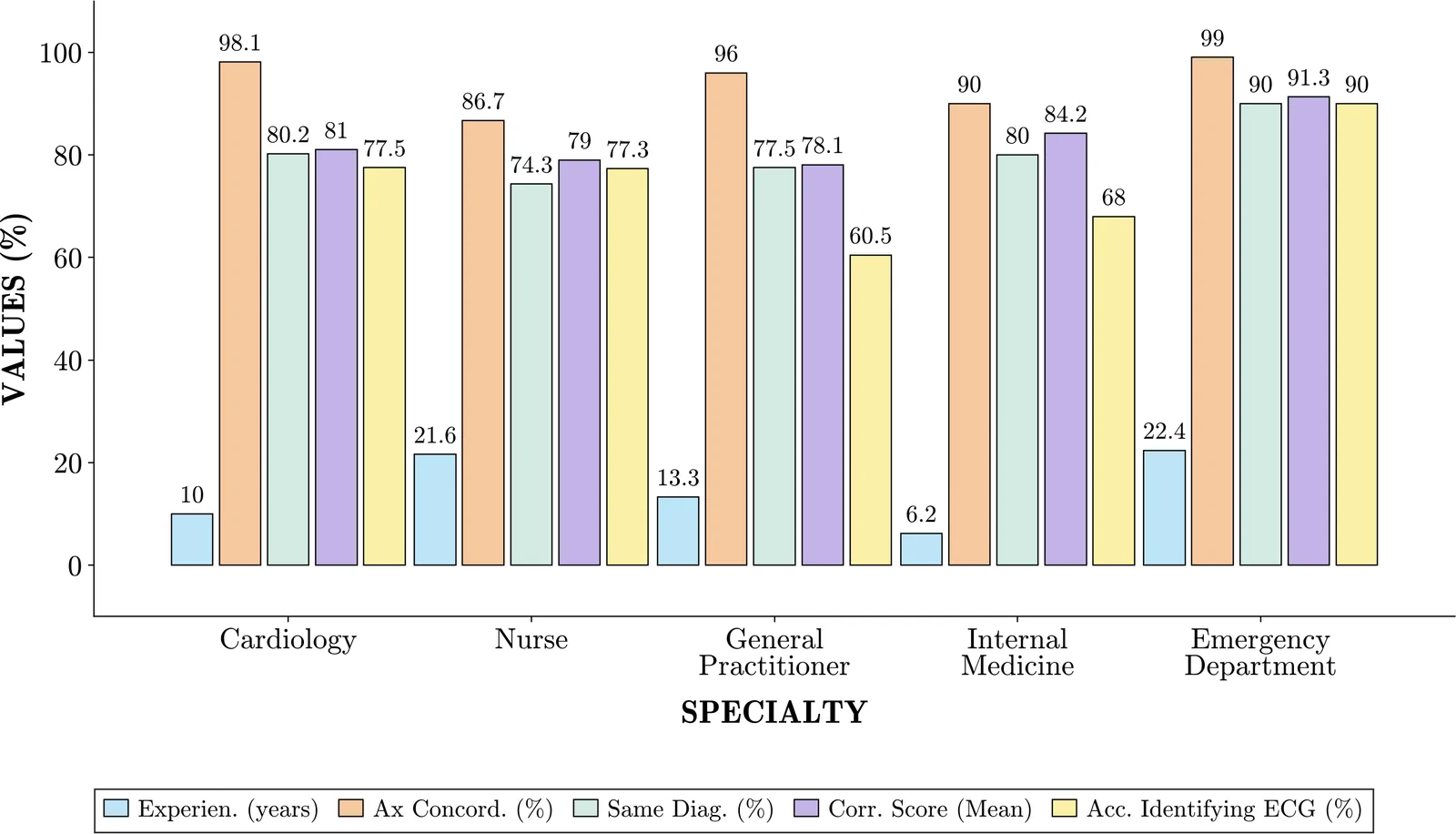
Comparison of metrics by specialty. ECG: electrocardiogram.

The correct identification of the source of the ECG (from a conventional source or from smartwatches) reaches an average of 75.6%. Emergency specialists show the highest accuracy (90%), followed by cardiologists (77.5%) and nurses (77.3%). General practitioners and internal medicine specialists show lower levels of accuracy (60.5% and 68%, respectively). As the similarity between the traces increases, the difficulty of correctly identifying the source of the ECG also increases. Among the 20 cases, one sinus rhythm ECG obtained from a healthy participant (case 2) is included as a control.

Before analyzing specific results, the cases were grouped by pathology to assess the behavior of the experimental device in different clinical scenarios. The thematic subgroups were not mutually exclusive; therefore, cases presenting more than one clinically relevant electrocardiographic abnormality could be included in more than one subgroup. Case 12, the only VT case in the cohort, was not included in the thematic subgroup analyses because no additional VT cases were available to constitute a homogeneous subgroup. It was included in the overall analysis and is presented individually in Multimedia Appendix 1 (Figure S13). In cases of ischemic heart disease (cases 6, 7, 13, 15, and 18), the concordance of the axis was high, with an average of 93.4%. Emergency specialists achieved 100%, while cardiologists reached 96.4%. Consistent diagnoses averaged 86.1%, with emergency specialists (96%) and cardiologists (87.2%) achieving the best results. The correlation scores had a mean of 84.9 (SD 19.2), with emergency physicians assigning the highest scores (95.2), followed by internal medicine specialists (90.0). The accuracy in identifying the source of the ECG was 76.0%, with emergency specialists standing out at 92% (Table S22 in Multimedia Appendix 1). In cases of pacemaker rhythms (cases 9, 10, and 14), the concordance of the axis was 94.5%; emergency specialists achieved 100% and cardiologists 98%. However, consistent diagnoses were lower, averaging 32.2%, and emergency specialists performed best at 46.7%. The correlation scores averaged 55.5 (SD 27.1), and internal medicine specialists achieved the highest scores (63.7). The accuracy in identifying the source of the ECG was high, averaging 93.7%, led by cardiologists (96.7%) and nurses (95.6%) (Table S23 in Multimedia Appendix 1).

The low correlation score between the tracings and the low percentage of identifying the same diagnosis were conditioned by the absence of pacemaker spikes in the smartwatch ECG tracing. In conventional electrocardiographs, a high-pass filter of 0.05 Hz and a low-pass filter of 150 Hz were configured, according to the recommendations of the ECG standardization and interpretation guidelines, published by the American Heart Association, the American College of Cardiology, and the Heart Rhythm Society [23]. The smartwatch, on the other hand, uses a more restrictive low-pass filter of around 30 to 45 Hz [24], which eliminates high-frequency signals such as pacemaker spikes without affecting the typical morphology of the pacemaker-stimulated ECG (Figure 6).

**Figure 6.**
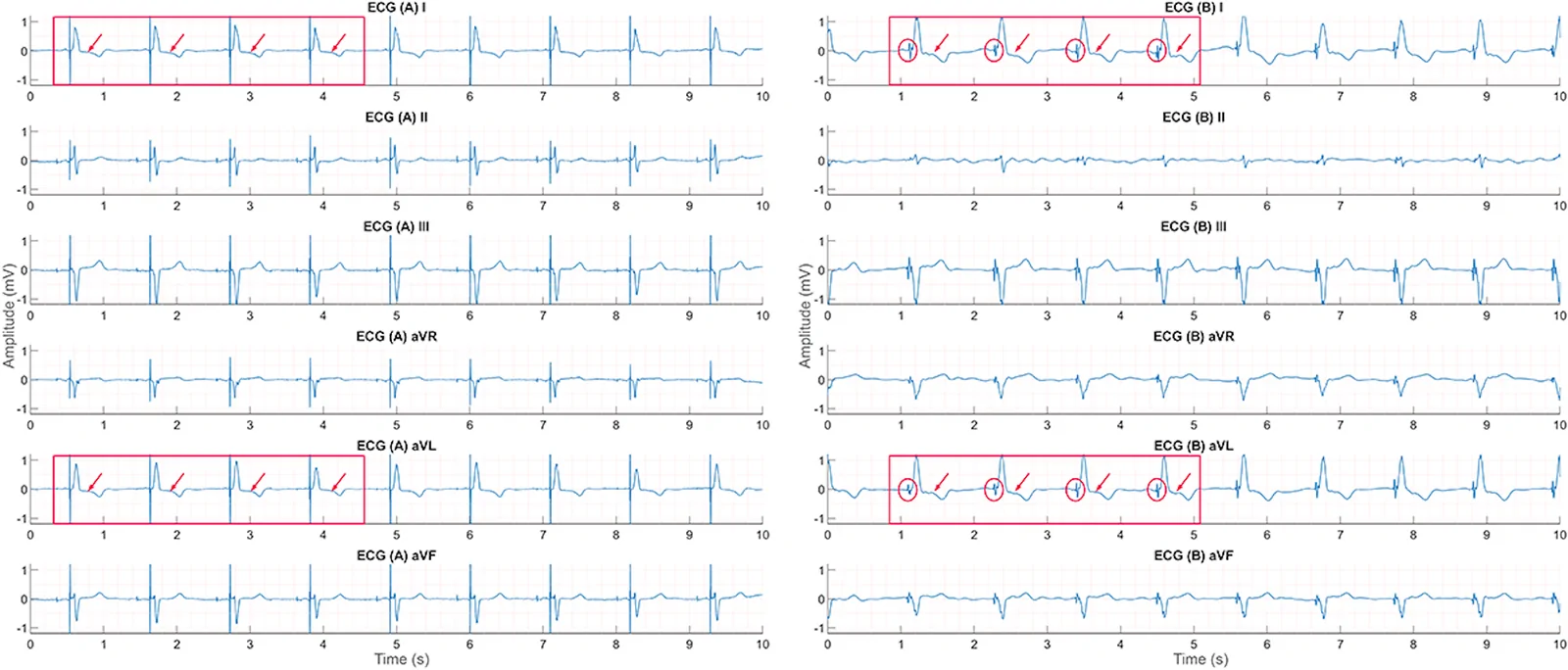
ECG A corresponds to the tracing obtained with a conventional electrocardiograph. In leads I and aVL, four cardiac cycles stand out, showing the inverted T-wave, a typical characteristic of the left bundle branch block pattern associated with pacemaker stimulation, with the ventricular electrode located in the right ventricle. On the other hand, ECG B is the tracing recorded by the smartwatch. In leads I and aVL, it is observed that it preserves the same morphology of left bundle branch block. The pacing spikes, which are circled to highlight them, appear attenuated due to the greater bandwidth restriction of the device. aVL: augmented voltage left; ECG: electrocardiogram.

To complement these findings, a quantitative comparison between conventional ECG (A) and smartwatch-derived ECG (B) was performed across all cases, and the results are summarized in Table 1 (details for each case are provided in Multimedia Appendix 1). Quantitative analysis demonstrated small mean differences between modalities, with particularly high agreement observed in structurally stable parameters such as heart rate (mean 77.2, SD 22.3 vs mean 75.9, SD 23.1 bpm; Δ=1.3 bpm) and PR interval (mean 176.2, SD 29.1 vs mean 180.8, SD 19.8 ms; Δ=−4.6 ms), computed only for cases in which each parameter was measurable. Greater variability was observed in time-dependent parameters such as QT (Δ=−19.2 ms) and QTc (Δ=−12.7 ms). For parameters representing atrial activation in eligible cases, the PP interval showed a mean of 859.0 (SD 175.5) ms vs 877.0 (SD 181.1) ms (Δ=−18.0 ms), while P-wave mean duration was 106.3 (SD 9.6) vs 119.6 (SD 13.9) ms (Δ=−13.3 ms). The electrical axis showed complete concordance between the two methods in all cases. Overall, structurally stable parameters such as heart rate and PR interval showed high agreement, whereas time-dependent parameters such as QT and QTc exhibited greater variability, with larger interval differences observed in some individual cases.

**Table 1.** Quantitative comparison of conventional electrocardiogram (ECG) (A) and smartwatch-derived ECG (B) across all cases in the cohort^a^.

Parameter	ECG A, mean (SD)	ECG B, mean (SD)	Mean difference (A − B)
Heart rate (bpm)	77.2 (22.3)	75.9 (23.1)	1.3
PR^b^ interval (ms)^c^	176.2 (29.1)	180.8 (19.8)	−4.6
QRS^d^ duration (ms)	105.2 (19.4)	117.2 (24.2)	−12.0
QT interval (ms)	417.1 (55.7)	436.3 (55.4)	−19.2
QTc^e^ (ms)	441.1 (32.5)	453.8 (33.2)	−12.7
RR^f^ interval (ms)	843.7 (283.8)	878.0 (351.4)	−34.3
PP^g^ interval (ms)^c^	859.0 (175.5)	877.0 (181.1)	−18.0
P-wave duration (ms)^c^	106.3 (9.6)	119.6 (13.9)	−13.3

a Data are presented as mean (SD). Mean differences were calculated as ECG A minus ECG B. The electrical axis showed complete concordance between the two methods in all cases.

b PR: P-R interval.

c Calculated exclusively from cases with physiologically present and measurable waveforms, to avoid zero-value calculation bias in nonapplicable conditions. Number of contributing cases (ECG A/ECG B): 12/11 (PR interval), 12/11 (PP interval), and 15/14 (P-wave duration). Where a parameter was measurable in one modality but not in the other, the case contributes only to the modality in which it could be determined; the mean difference therefore represents the difference between the modality-specific means. Exclusions were applied as follows: (1) PR interval (ECG A, n=12; ECG B, n=11) was excluded owing to absent or unmeasurable atrioventricular conduction in complete AV block (case 1), second-degree AV block 2:1 (case 4), atrial fibrillation/flutter (cases 5, 17, 19, and 20), ventricular tachycardia (case 12), and second-degree AV block Mobitz I (case 18); additionally, it was excluded from ECG B only in the ventricularly paced rhythm with nondiscernible P waves (case 9, ECG B only). (2) PP interval (ECG A, n=12; ECG B, n=11) was excluded owing to absent, independent, or highly irregular/fibrillatory atrial rhythms in complete AV block (case 1), second-degree AV block 2:1 (case 4), atrial fibrillation/flutter (cases 5, 17, 19, and 20), ventricular tachycardia (case 12), and second-degree AV block Mobitz I (case 18); additionally, it was excluded from ECG B only in the ventricularly paced rhythm (case 9, ECG B only). (3) P-wave duration (ECG A, n=15; ECG B, n=14) was excluded owing to absent or nondiscernible P waves in atrial fibrillation/flutter (cases 5, 17, 19, and 20) and ventricular tachycardia (case 12); additionally, it was excluded from ECG B only in the ventricularly paced rhythm with nondiscernible P waves (case 9, ECG B only).

d QRS: QRS complex.

e QTc: corrected QT interval.

f RR: R-R interval.

g PP: P-P interval.

In cases of atrioventricular or bundle branch blocks (cases 1, 3, 4, 6, 8, 11, 16, and 18), the concordance of the axis averaged 93.5%, with emergency specialists at 97.5% and cardiologists at 96.8%. Consistent diagnoses reached 84.4%, with emergency specialists standing out at 97.5% and cardiologists at 87.2%. The correlation score averaged 82.6 (SD 20.0), and emergency specialists granted the highest scores (96.8%). The accuracy in identifying the source of the ECG was 71.2%, with emergency specialists placed at 85.0% (Table S24 in Multimedia Appendix 1).

In cases of atrial arrhythmias (cases 5, 17, 19, and 20), the concordance of the axis was averaged at 96.8%, with emergency specialists achieving 100% and cardiologists 99.5%. Consistent diagnoses averaged 86.8%, with emergency specialists (100%) and internal medicine specialists (95.0%) showing the highest agreement. The correlation scores averaged 86.1 (SD 17.3), and emergency specialists granted the highest score (99.8%). Accuracy in identifying the source of the ECG was 69.7%, with emergency specialists achieving 90% (Table S25 in Multimedia Appendix 1).

Ischemic heart disease, atrial arrhythmias, and atrioventricular or bundle branch blocks demonstrated the highest diagnostic concordance. Conversely, pacemaker rhythms exhibited challenges for intermethod correlation and consistency (Figure 6). Emergency physicians consistently achieved superior diagnostic accuracy.

## Discussion

### Principal Findings

This study validated the reconstruction of 12-lead ECGs from smartwatch-recorded signals by aligning leads and generating augmented leads. Our findings demonstrate high concordance between smartwatch-derived ECGs and conventional ECGs, particularly in ischemic heart disease and bundle branch blocks, supporting their agreement with conventional ECGs and their potential as a complementary tool for cardiac condition evaluation.

In addition, quantitative analysis of electrocardiographic parameters demonstrated small mean differences between modalities, with particularly high agreement in structurally stable measurements such as heart rate and PR interval, which supports the temporal consistency of the reconstructed signals despite sequential acquisition.

The alignment of leads and the generation of augmented leads proved feasible and effective, yielding clinically meaningful results. It is important to emphasize that the methodological innovation of this study primarily concerns the reconstruction of frontal plane leads, while precordial leads are directly acquired and subsequently synchronized; therefore, clinical validation was focused on the frontal plane, where the core methodological contribution lies. This innovative approach, using sequential tracings and advanced signal processing, overcomes previously identified technological barriers by enabling reconstruction of augmented leads with clinically meaningful electrocardiographic information.

### Comparison With Prior Work

Previous studies have explored alternative strategies for extending smartwatch ECG capabilities, including beat averaging to generate representative ECG complexes [11,12], direct sequential comparison of smartwatch-acquired leads without interlead synchronization [13], and learning-based generative reconstruction methods designed to synthesize missing ECG leads from partial input signals [14].

In contrast, our approach preserves beat-level morphology by aligning RR intervals across sequential recordings and analytically deriving the augmented leads, rather than generating synthetic electrocardiographic information. This distinction is particularly relevant because smartwatch recordings are inherently asynchronous, creating a specific methodological challenge that differs substantially from conventional ECG reconstruction settings.

In the case of learning-based generative models [14], the objective is typically to reconstruct a full 12-lead ECG from one or more conventional ECG leads by solving a data-driven synthesis problem. Since these reconstructed signals are synchronized with the original acquisition, waveform similarity metrics can be used to assess reconstruction fidelity. However, the applicability of this methodology to wearable devices remains uncertain, as real-world smartwatch acquisition introduces additional constraints that are not typically represented in existing experimental frameworks.

An important distinction is that lead I, as defined in conventional electrocardiography, is not directly available on wearable devices due to physical hardware constraints, as the electrodes are positioned in very close proximity to each other. Therefore, the starting signal obtained from a smartwatch represents only an approximation of conventional lead I rather than a true equivalent.

Our methodology differs conceptually in that it operates directly on actual patient-recorded smartwatch signals, using RR-block resampling to address the challenge of sequential, asynchronous acquisition while preserving temporal consistency across leads. Rather than reconstructing an approximation from synthetic data, the proposed framework builds the 12-lead ECG from real electrocardiographic measurements acquired under wearable conditions.

### Clinical Implications

The increasing commercial availability and adoption of smartwatches have positioned these devices as potentially valuable tools in cardiovascular medicine. While their current clinical utility is primarily established in atrial fibrillation detection through single-lead ECG acquisition [25], the inherent limitation of a single lead restricts their applicability in broader diagnostic scenarios, such as ischemic heart disease, myocardial pathology, or accurate arrhythmia localization.

The methodology presented in this study expands this diagnostic scope by enabling reconstruction of a 12-lead ECG from smartwatch-acquired signals. This aligns with growing interest in smartwatch-based cardiac assessment for ischemic heart disease [7], including our group’s prior work demonstrating the feasibility of multilead smartwatch ECG acquisition [6,26]. Our group is also currently conducting a large-scale multicenter study to evaluate an abbreviated 3-lead smartwatch algorithm for ischemic heart disease detection [27].

Our analysis further showed that emergency physicians and cardiologists ranked first and second, respectively, in accuracy in identifying the ECG source and in axis concordance. For the correlation score, however, emergency physicians ranked highest, followed by internal medicine specialists, with cardiologists in third place. This finding highlights the importance of clinical expertise in interpreting complex electrocardiographic tracings and suggests that smartwatch-derived ECGs may have practical value as diagnostic adjuncts when integrated into established clinical workflows.

### Future Directions

It should be noted that the reconstructed ECGs in this study were not presented in a standard clinical display format, as the primary objective was to demonstrate the feasibility of the proposed methodology as a proof-of-concept. The visualization layout used represents an initial approximation for comparative purposes; however, the underlying signal processing framework is flexible and can be adapted to conventional ECG display formats, including those incorporating a continuous rhythm strip. Future developments will focus on implementing standardized clinical layouts to enhance interpretability and practical applicability.

Beyond technical implementation, further research is needed to evaluate the clinical applicability of smartwatch-derived 12-lead ECG reconstruction in larger and pathology-specific cohorts. Smartwatch-based ECG technologies may also prove useful in broader remote cardiac monitoring scenarios, including rhythm surveillance and bradyarrhythmia detection, as suggested by previously published case reports [28]. Broader validation will be essential to determine performance across different cardiovascular conditions and real-world monitoring settings.

Future integration with AI may further enhance wearable ECG analysis through individualized longitudinal monitoring, automated anomaly detection, and decision-support tools [29]. These applications remain exploratory and require rigorous clinical validation before adoption in routine practice.

### Limitations

Despite the promising results, several limitations warrant consideration, particularly given the proof-of-concept nature of this study. First, the current methodology involves a manual and potentially subjective visual review for optimal segment selection from smartwatch recordings. However, this is a known challenge with clear pathways for automation through signal quality indices in future iterations. Similarly, while a learning-based R peak detector was used [19], complex pathologies, including pacemaker rhythms, occasionally required manual review due to inherent challenges in interpretation and consistency. This reliance on manual intervention, although appropriate for early-stage validation, may limit scalability and real-world applicability, highlighting the need for fully automated signal processing workflows in future implementations. These issues, common in early-stage development, underscore the need for optimized recording techniques and improved filtering in portable devices, rather than representing insurmountable barriers.

Second, the sequential nature of smartwatch ECG acquisition inherently limits the applicability of conventional signal fidelity metrics, such as Pearson correlation coefficients, root mean square error, or signal-to-noise ratio. Unlike standard ECG systems, where all leads are recorded simultaneously, smartwatch-derived leads are obtained from different cardiac cycles and subsequently aligned through resampling. Therefore, direct point-by-point waveform comparison would not reflect true reconstruction accuracy but rather physiological beat-to-beat variability and temporal misalignment. For this reason, the present study focused on clinically meaningful interval-based comparisons and expert interpretation, which better reflect real-world diagnostic applicability.

Third, sequential acquisition may result in recordings being obtained during different phases of the respiratory cycle, which can alter the anatomical position of the heart and lead to variations in the electrical axis and QRS amplitude. Consequently, the application of the Goldberger equations to signals acquired under different respiratory conditions may introduce discrepancies compared to true simultaneous recordings.

Fourth, the limited frequency bandwidth of current smartwatch ECG devices represents an additional technical constraint, particularly for the detection of high-frequency components such as pacemaker spikes. Commercially available smartwatches typically operate within a reduced bandwidth (approximately 0.5‐40 Hz), compared to conventional electrocardiographs (0.05‐150 Hz), due to the application of relatively aggressive filtering, which may attenuate high-frequency signals and contribute to reduced diagnostic consistency in specific scenarios. This limitation specifically affects the identification of pacing artifacts, although the overall morphology of paced rhythms is generally preserved.

Fifth, precordial leads obtained with the smartwatch should be interpreted as approximations rather than as true unipolar recordings due to the absence of a WCT, which represents an inherent electrophysiological limitation of the device.

Sixth, the validation was conducted on a cohort of 20 cases, including patients with different cardiac conditions and one healthy control. Although the cohort included a range of electrocardiographic findings, some conditions were represented by more than one case (eg, atrial fibrillation and atrial flutter). While this limits generalizability, it is appropriate for a proof-of-concept study focused on validating the proposed methodology for 12-lead ECG reconstruction. In this context, the primary aim of the study was to demonstrate the technical feasibility and preliminary clinical agreement of the proposed reconstruction methodology under controlled conditions, rather than to establish diagnostic performance across specific pathologies. Therefore, larger studies including a greater number of patients within each diagnostic category will be necessary to further evaluate the clinical applicability and generalizability of the proposed methodology.

Seventh, the study cohort was intentionally composed of patients with evident electrocardiographic abnormalities, which may introduce a degree of selection bias and limit the extrapolation of results to real-world populations with varying pretest probabilities.

Therefore, the findings should not be interpreted as reflecting predictive or screening performance in unselected real-world populations, particularly in settings with lower pretest probability or more subtle electrocardiographic alterations.

Eighth, although the validation relied on expert opinion, a clinical electrophysiologist served as the “gold standard” to guide ECG interpretation, thereby acknowledging the inherent subjective component of ECG reading. To further strengthen these findings, more standardized and objective validation methods are needed in subsequent research.

Ninth, the use of anonymized survey responses may introduce response bias, and the absence of variability observed in certain subgroups should be interpreted with caution, as individual responses could not be verified. Furthermore, differences in subgroup sizes across specialties may influence the aggregated results, and although weighted analyses were used, residual imbalances may persist.

Tenth, to fully establish clinical utility, larger-scale studies focusing on specific pathologies are essential. Each cardiac condition presents unique diagnostic considerations, and the impact of early diagnosis varies significantly, from life-saving interventions in some cases to less critical implications in others. Therefore, future research should meticulously investigate the performance of smartwatch-derived ECGs across diverse cardiac conditions to ensure their reliability and appropriate clinical application.

### Conclusions

This proof-of-concept study demonstrates the technical feasibility of reconstructing a synchronized 12-lead ECG from smartwatch-acquired signals through sequential lead alignment and analytical generation of augmented leads.

The proposed methodology showed preliminary clinical agreement with conventional ECGs, particularly in cases of ischemic heart disease and bundle branch blocks, while preserving beat-level morphology across sequential recordings.

These findings support the potential applicability of smartwatch-derived multilead ECG reconstruction as a complementary approach for wearable cardiac monitoring under controlled acquisition conditions.

## Supplementary material

10.2196/86621 Multimedia Appendix 1 Detailed clinical case analyses, representative ECG figures, quantitative electrocardiographic measurements, the signal-processing script used for augmented limb lead generation, and survey results.

## Abbreviations

AF: atrial fibrillation
aVF: augmented voltage foot
aVL: augmented voltage left
aVR: augmented voltage right
ECG: electrocardiogram
HRV: heart rate variability
QTc: corrected QT interval
VT: ventricular tachycardia
WCT: Wilson Central Terminal

## References

[1] Samol A, Bischof K, Luani B, Pascut D, Wiemer M, Kaese S (2019). Single-lead ECG recordings including Einthoven and Wilson leads by a smartwatch: a new era of patient directed early ECG differential diagnosis of cardiac diseases?. Sensors (Basel).

[2] Perez MV, Mahaffey KW, Hedlin H (2019). Large-scale assessment of a smartwatch to identify atrial fibrillation. N Engl J Med.

[3] Lubitz SA, Faranesh AZ, Selvaggi C (2022). Detection of atrial fibrillation in a large population using wearable devices: the Fitbit Heart Study. Circulation.

[4] Campo D, Elie V, de Gallard T (2022). Atrial fibrillation detection with an analog smartwatch: prospective clinical study and algorithm validation. JMIR Form Res.

[5] Barrera N, Solorzano M, Jimenez Y, Kushnir Y, Gallegos-Koyner F, Dagostin de Carvalho G (2025). Accuracy of smartwatches in the detection of atrial fibrillation: a systematic review and diagnostic meta-analysis. JACC Adv.

[6] Buelga Suárez M, Pascual Izco M, Pastor Pueyo P, Lozano Granero C, García Montalvo J, Alonso Salinas GL (2023). Smartwatch ECG tracing and ischemic heart disease: ACS Watch Study. Cardiology.

[7] Spaccarotella CAM, Polimeni A, Migliarino S (2020). Multichannel electrocardiograms obtained by a smartwatch for the diagnosis of ST-segment changes. JAMA Cardiol.

[8] Guarnieri G, Mapelli M, Moltrasio M, Agostoni P, Tondo C (2024). Connected health: ventricular tachycardia detection with Apple Watch—a case report. Heliyon.

[9] Burke J, Haigney MCP, Borne R, Krantz MJ (2020). Smartwatch detection of ventricular tachycardia: case series. HeartRhythm Case Rep.

[10] Madias JE (2013). A proposal for a 9-lead electrocardiogram recorded via the Wilson’s central terminal. Ann Noninvasive Electrocardiol.

[11] Ugander M, Curtis T (2023). A method and a system for producing a standard 12-lead ECG. https://patents.google.com/patent/US20230363684A1/en?oq=US20230363684A1.

[12] Albert DE (2016). Two electrode apparatus and methods for twelve lead ECG. https://patents.google.com/patent/US9351654B2/en?oq=US9351654B2.

[13] van der Zande J, Strik M, Dubois R (2023). Using a smartwatch to record precordial electrocardiograms: a validation study. Sensors (Basel).

[14] Chen J, Wu W, Liu T, Hong S (2024). Multi-channel masked autoencoder and comprehensive evaluations for reconstructing 12-lead ECG from arbitrary single-lead ECG. NPJ Cardiovasc Health.

[15] Barold SS (2003). Willem Einthoven and the birth of clinical electrocardiography a hundred years ago. Card Electrophysiol Rev.

[16] Avila CO (2019). Novel use of Apple Watch 4 to obtain 3-lead electrocardiogram and detect cardiac ischemia. Perm J.

[17] Gamble P, McManus H, Jensen D, Froelicher V (1984). A comparison of the standard 12-lead electrocardiogram to exercise electrode placements. Chest.

[18] (2011). PageWriter TC20/TC30 electrocardiographs: instructions for use [Report in Spanish]. https://blogs.hsjda.es/electromedicina/wp-content/uploads/sites/10/2019/04/03-01-094-677-0X-C00001-USO-ECG-Spanish_TC20_TC30_IFU.pdf.

[19] Plaza-Seco C, Barner KE, Holgado-Cuadrado R, Melgarejo-Meseguer FM, Rojo-Álvarez JL, Blanco-Velasco M (2025). Detection of intra-beat waves on ambulatory ECG using manifolds: an explainable deep learning approach. Biomed Signal Process Control.

[20] Sörnmo L, Laguna P (2005). Bioelectrical Signal Processing in Cardiac and Neurological Applications.

[21] Oppenheim AV, Schafer RW (2021). Discrete-Time Signal Processing.

[22] Madias JE (2008). On recording the unipolar ECG limb leads via the Wilson’s vs the Goldberger’s terminals: aVR, aVL, and aVF revisited. Indian Pacing Electrophysiol J.

[23] Kligfield P, Gettes LS, Bailey JJ (2007). Recommendations for the standardization and interpretation of the electrocardiogram: part I: the electrocardiogram and its technology: a scientific statement from the American Heart Association Electrocardiography and Arrhythmias Committee, Council on Clinical Cardiology; the American College of Cardiology Foundation; and the Heart Rhythm Society: endorsed by the International Society for Computerized Electrocardiology. Circulation.

[24] Knecht S, Waldmann F, Kuhn R (2023). Technical characterization of single-lead ECG signals from 4 different smartwatches and its potential clinical implications. JACC Clin Electrophysiol.

[25] Hindricks G, Potpara T, Dagres N (2021). 2020 ESC Guidelines for the diagnosis and management of atrial fibrillation developed in collaboration with the European Association for Cardio-Thoracic Surgery (EACTS): the Task Force for the diagnosis and management of atrial fibrillation of the European Society of Cardiology (ESC) developed with the special contribution of the European Heart Rhythm Association (EHRA) of the ESC. Eur Heart J.

[26] Buelga Suárez M, García Montalvo J, Alonso Salinas GL (2022). Smartwatches and ischemic heart disease. A future possibility?. Rev Esp Cardiol (Engl Ed).

[27] Buelga Suárez M, Pascual Izco M, García Montalvo J, Alonso Salinas GL (2024). Accuracy of smartwatch electrocardiographic recording in the acute coronary syndrome setting: rationale and design of the ACS WATCH II Study. J Clin Med.

[28] Buelga Suárez ML, Ramírez Martín J, Alonso Salinas GL (2023). Smartwatch health monitoring in the context of a complete heart block: a challenge beyond these watches’ capabilities. Emergencias.

[29] Lim J, Park G, Lee HS (2026). ECG trained artificial intelligence for the detection of patients with inducible myocardial ischemia. Eur Heart J Digit Health.

