# Supplementary material for: Generation of 12-Lead Electrocardiogram Using Smartwatches in Patients With Cardiac Conditions: Clinical Validation Study
Source: JMIR Cardio. 2026 Nov 5;10:e86621. doi: 10.2196/86621 (PMC13739267; doi:10.2196/86621)
Supplement: Supplementary file 1 [file cardio-v10-e86621-s001.pdf]

Appendix 1 SUPPLEMENTAL MATERIAL

Article Title: Generation of 12-Lead ECG Using Smartwatches in Patients with Cardiac Conditions: A Clinical Validation Study

Journal: JMIR Cardio

Authors: Mauro Buelga Suárez[1,2,3], Carmen Plaza-Seco[4], Roberto Matía Francés[3], Gonzalo Luis Alonso Salinas[5,6,7,*], Manuel Blanco-Velasco[4]

Affiliations:
[1] Cardiology Department, Hospital Universitario Ramón y Cajal, Madrid, 28034, Spain
[2] Medicine and Health Sciences Department, University of Alcalá, Alcalá de Henares, Madrid, 28805, Spain
[3] Ramón y Cajal Institute for Health Research (IRYCIS), Madrid, 28034, Spain
[4] Department of Signal Theory and Communications, University of Alcalá, Alcalá de Henares, Madrid, 28805, Spain
[5] Health Sciences Department, Universidad Pública de Navarra, Pamplona, Spain.
[6] Cardiology Department, Hospital Universitario de Navarra, Pamplona, Navarra, 31008, Spain
[7] Navarrabiomed (Miguel Servet Foundation), Instituto de Investigación Sanitaria de Navarra (IdiSNA), Pamplona, Spain.

Corresponding Author*: Gonzalo Luis Alonso (gonzalo.alonso.salinas@unavarra.es)

Non-standard Acronyms and Abbreviations (Figures)

AV: Atrioventricular
SR: Sinus rhythm
RBBB: Right Bundle Branch Block
AF: Atrial Fibrillation
LAFB: Left Anterior Fascicular Hemiblock
VT: Ventricular Tachycardia
STEMI: ST-Elevation Myocardial Infarction
LBBB: Left Bundle Branch Block

Clinical Case Analyses

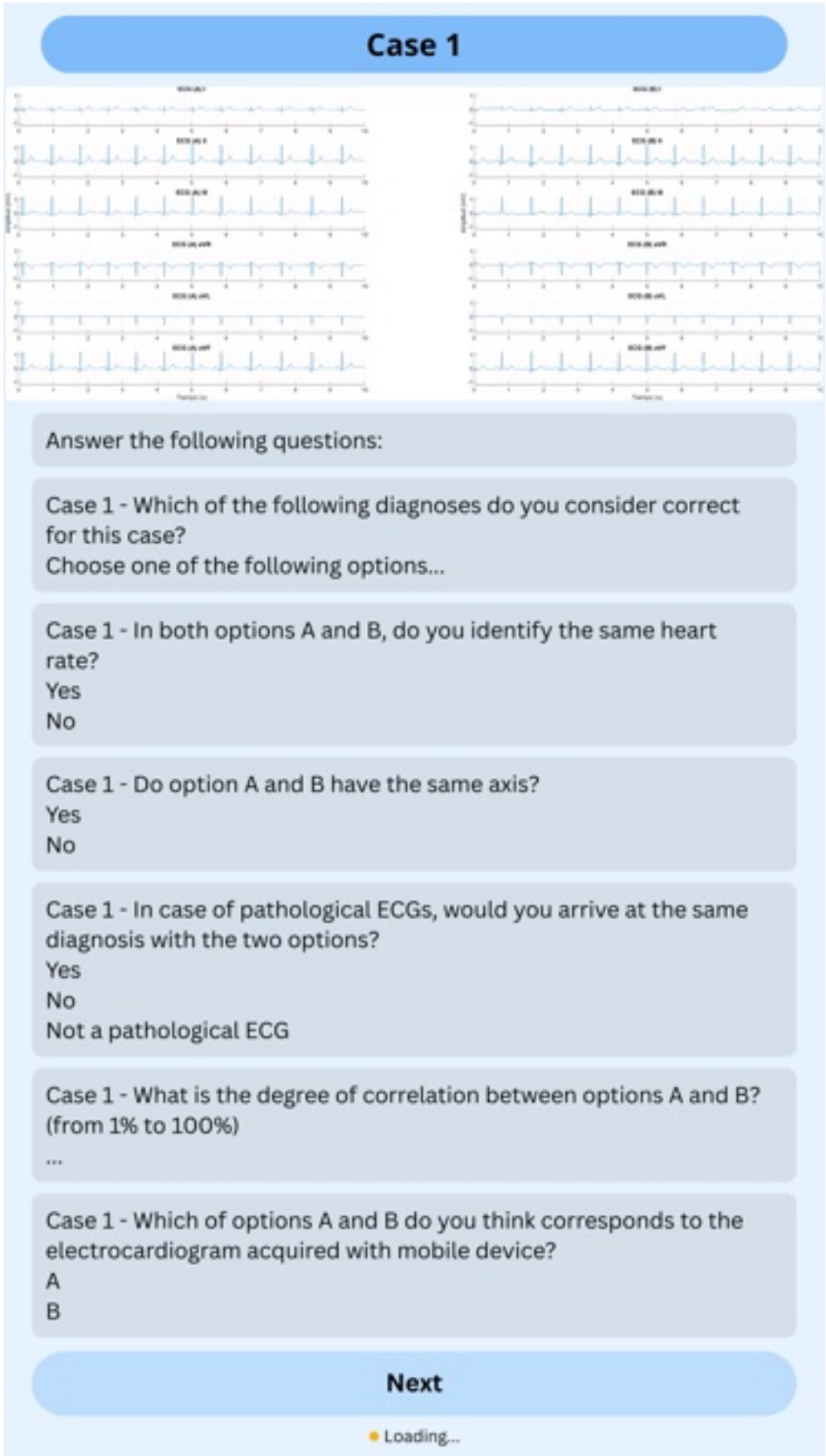

Fig. S1: A simple and anonymous survey designed to subjectively analyze the concordance between both electrocardiograms

To facilitate interpretation, in all tracings the A panels correspond to the conventional ECG, and the B panels correspond to the smartwatch ECG. This organization is intended to assist the reader in comparing both modalities. Presented below are the clinical cases analyzed by the clinical participants during validation, together with the expert arrhythmologist's assessment. In addition, a quantitative comparison of electrocardiographic parameters between both tracings is provided for each case, allowing an objective analysis of agreement between conventional and smartwatch-derived ECGs. All quantitative measurements were performed by experienced cardiology researchers using standardized digital ECG analysis tools, ensuring consistent assessment of intervals and waveform characteristics across both modalities.

Case 1

In panel A, atrioventricular (AV) dissociation is observed, with P waves occurring at a higher frequency than the narrow and rhythmic QRS complexes. This is diagnostic of complete AV block with a junctional escape rhythm. For a correct diagnosis, it is essential to identify atrial activity at a frequency higher than the ventricular rhythm. In panel B, the P-wave duration is relatively longer compared to its amplitude, giving it a somewhat blunted appearance. The junctional escape rhythm is slightly slower. This change in P-wave morphology makes the diagnosis more challenging, as it hampers recognition of atrial activity, particularly when it coincides with ventricular repolarization (T wave).

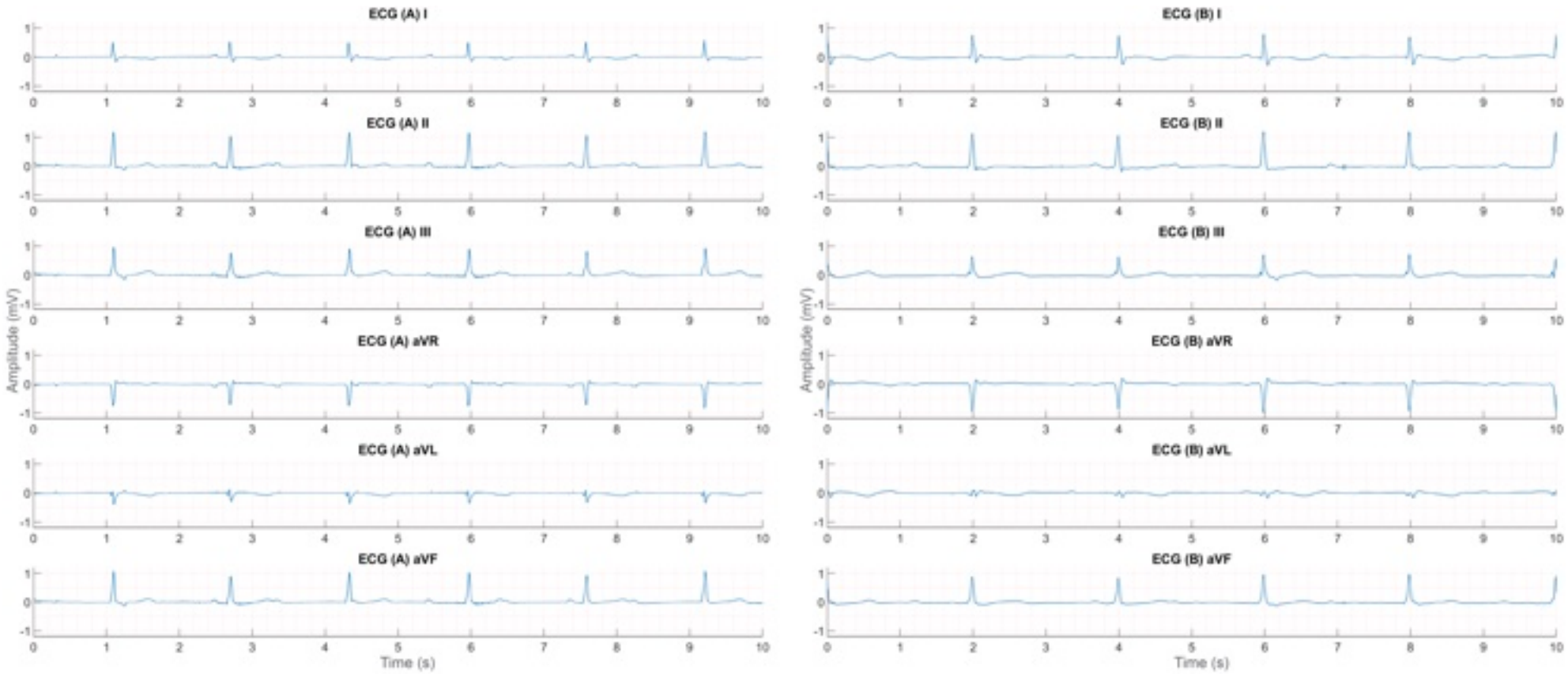

Fig. S2: Case 1. Complete AV block

Case 1. Conventional ECG (A) vs Smartwatch ECG (B)

Parameter	ECG A	ECG B	Difference (A-B)
Heart Rate (bpm)	37	30	7
PR interval (ms)	-	-	-
QRS duration (ms)	111	129	-18
QT interval (ms)	563	544	19
QTc (ms)	468	391	77
RR interval (ms)	1617	1998	-381
P wave duration (ms)	93	103	-10
PP interval (ms)	-	-	-

Case 2

In panel A, a normal sinus rhythm is seen (positive P wave in leads I, II, III and negative in aVR). In panel B, although both the P and T waves display a somewhat rounder morphology (less pointed), the same diagnosis can easily be reached. The QRS interval and ventricular repolarization remain broadly comparable despite moderate differences in interval duration.

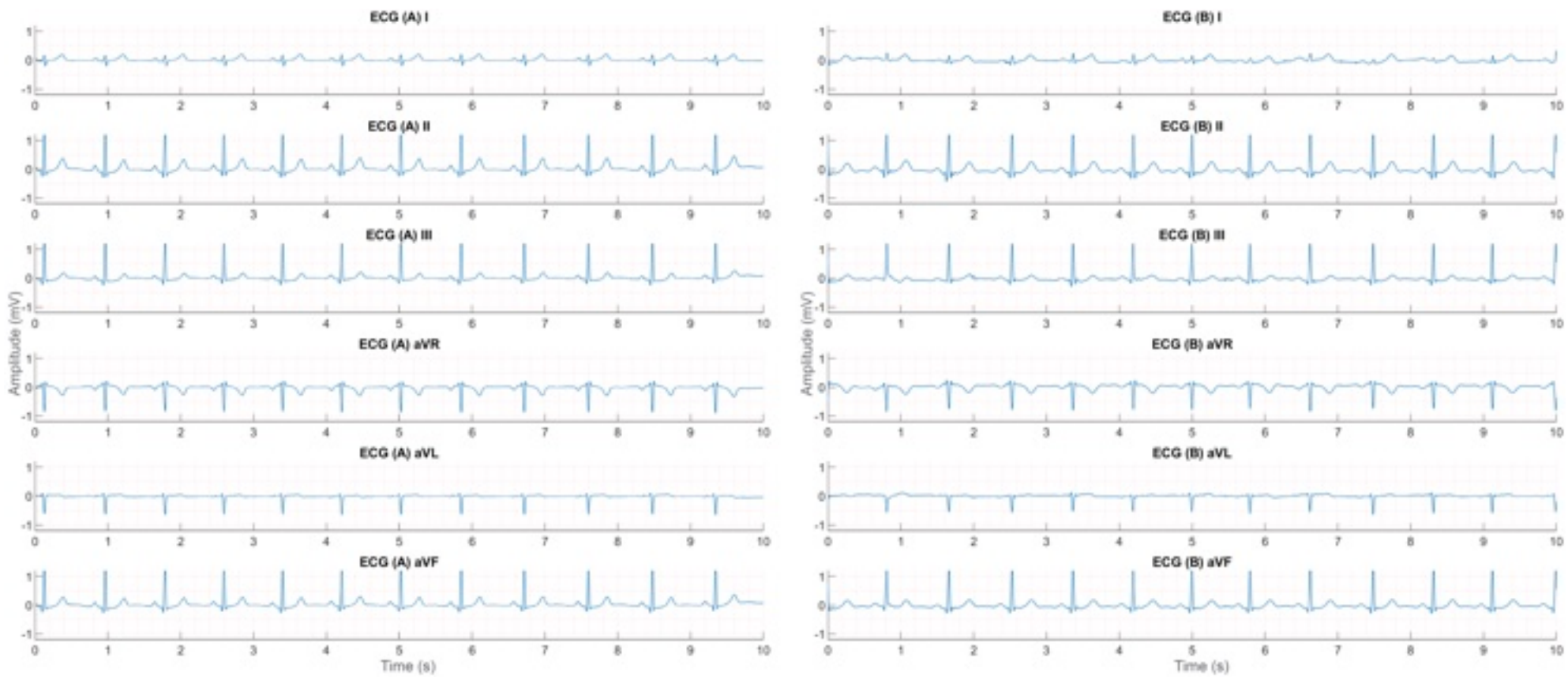

Fig. S3: Case 2. SR

Case 2. Conventional ECG (A) vs Smartwatch ECG (B)

Parameter	ECG A	ECG B	Difference (A-B)
Heart Rate (bpm)	71	71	0
PR interval (ms)	160	184	-24
QRS duration (ms)	82	97	-15
QT interval (ms)	386	421	-35
QTc (ms)	412	446	-34
RR interval (ms)	833	836	-3
P wave duration (ms)	108	133	-25
PP interval (ms)	833	836	-3

Case 3

In panel A, a sinus P wave (positive in leads I, II, III) is observed, but it appears notched in the inferior leads (II, III, aVF) with a duration slightly greater than 120 ms, suggesting intra-atrial conduction disturbance. The PR interval exceeds 230 ms, consistent with first-degree AV block. In panel B, the notching of the P wave in the inferior leads is less evident, but the PR interval remains equally prolonged, allowing the same diagnosis of first-degree AV block.

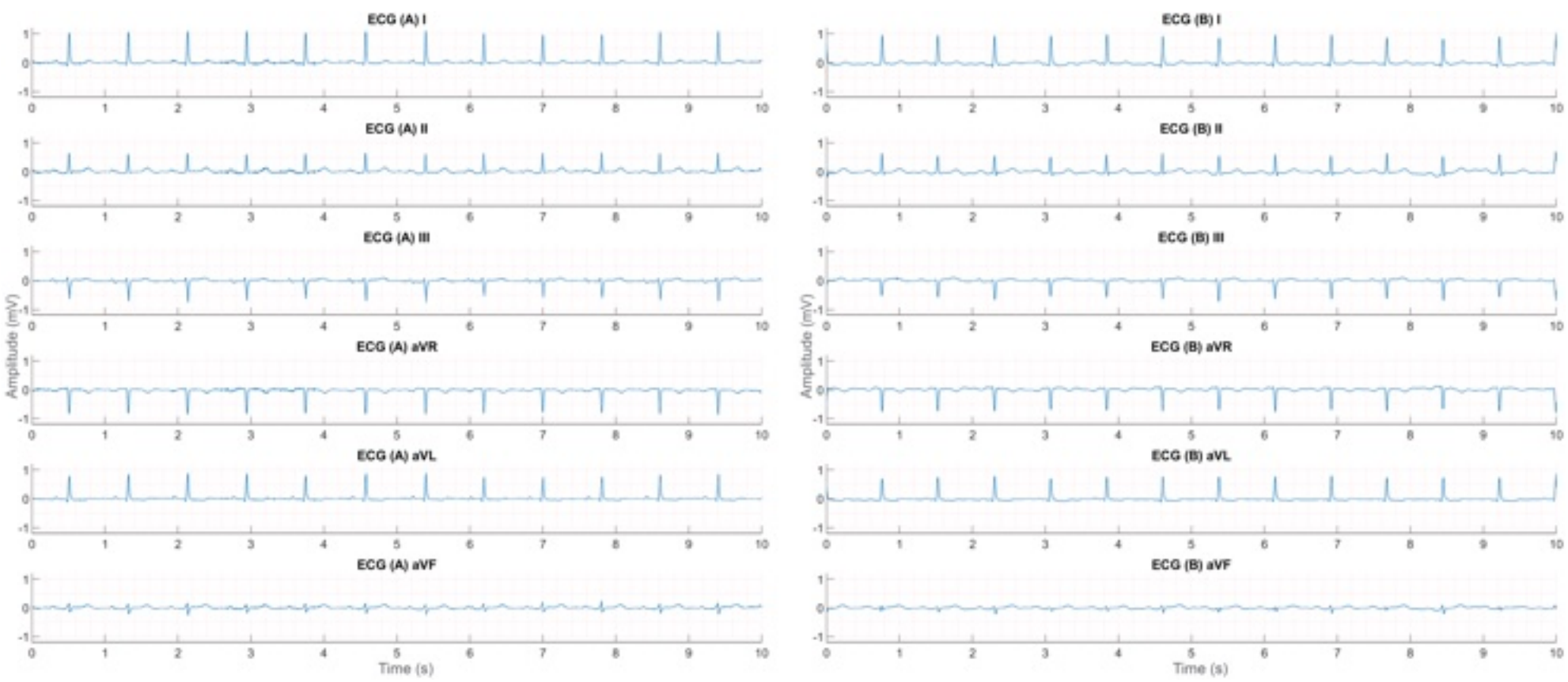

Fig. S4: Case 3. First-degree AV block

Case 3. Conventional ECG (A) vs Smartwatch (B)

Parameter	ECG A	ECG B	Difference (A-B)
Heart Rate (bpm)	74	78	-4
PR interval (ms)	230	212	18
QRS duration (ms)	93	97	-4
QT interval (ms)	398	400	-2
QTc (ms)	428	435	-7
RR interval (ms)	807	769	38
P wave duration (ms)	111	126	-15
PP interval (ms)	807	769	38

Case 4

In panel A, P waves are seen at a higher frequency than the QRS complexes, with two P waves for each QRS and a constant PR interval for the conducted beats. This is diagnostic of second-degree AV block with 2 : 1 conduction. The QRS morphology shows left axis deviation due to left anterior fascicular block (S wave in III > II), with an R' pattern in aVR and broad S waves in lead I, consistent with right bundle branch block. The near absence of R waves in the inferior leads further suggests inferior necrosis. In panel B, the P waves are blunter, and every second P wave is not clearly visible as it coincides with the T wave, preventing a reliable diagnosis of AV block. QRS complexes are markedly wider and of greater amplitude, and the absence of a small initial R wave in lead II strengthens suspicion of inferior necrosis.

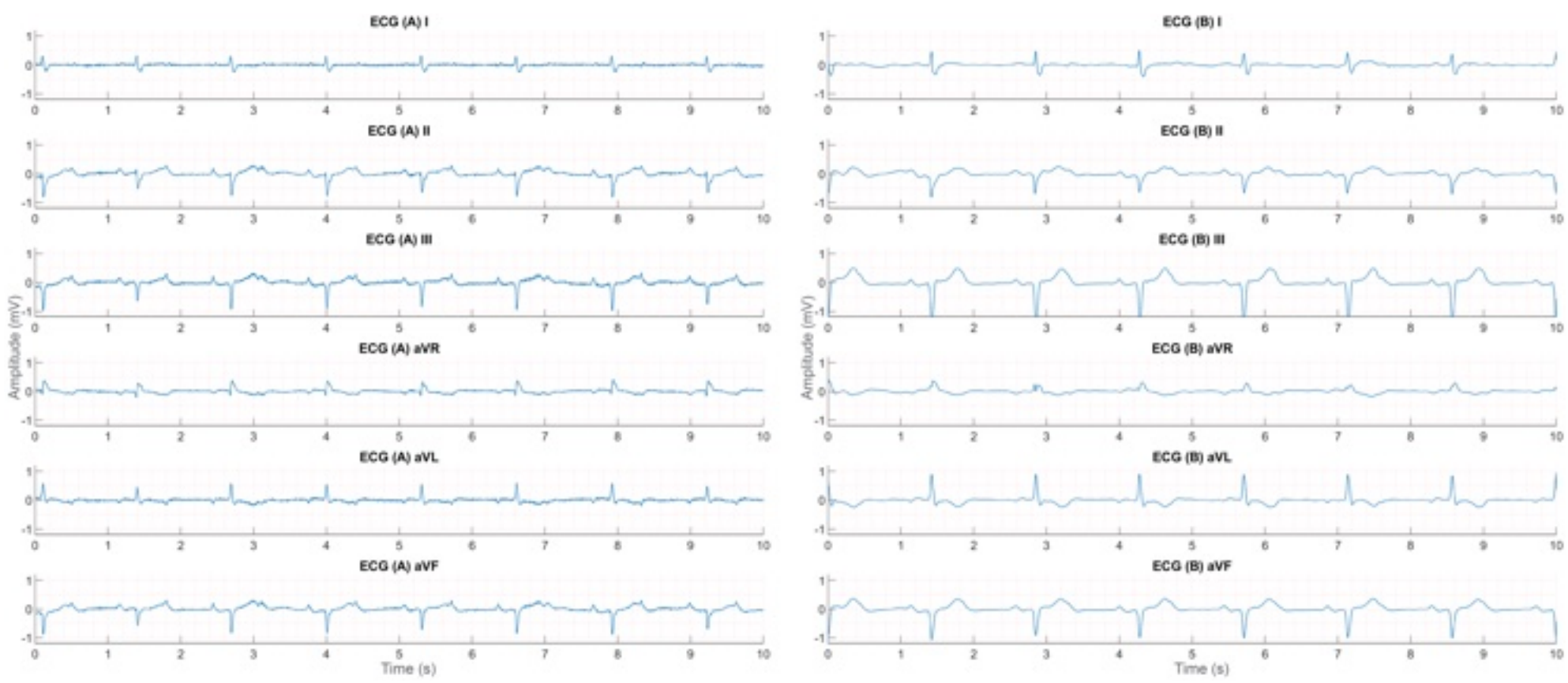

Fig. S5: Case 4. Second-degree AV block (2:1) with RBBB

Case 4. Conventional ECG (A) vs Smartwatch (B)

**Parameter**	**ECG A**	**ECG B**	**Difference (A-B)**
Heart Rate (bpm)	45	42	3
PR interval (ms)	-	-	-
QRS duration (ms)	103	138	-35
QT interval (ms)	504	545	-41
QTc (ms)	456	480	-24
RR interval (ms)	1309	1425	-116
P wave duration (ms)	105	135	-30
PP interval (ms)	-	-	-

Case 5

In panel A, the characteristic f waves of atrial fibrillation are clearly visible, together with the absence of organized atrial activity (no P waves) and an irregularly irregular ventricular rhythm, confirming the diagnosis. In panel B, although the atrial fibrillatory activity is less evident, the same diagnostic criteria are met.

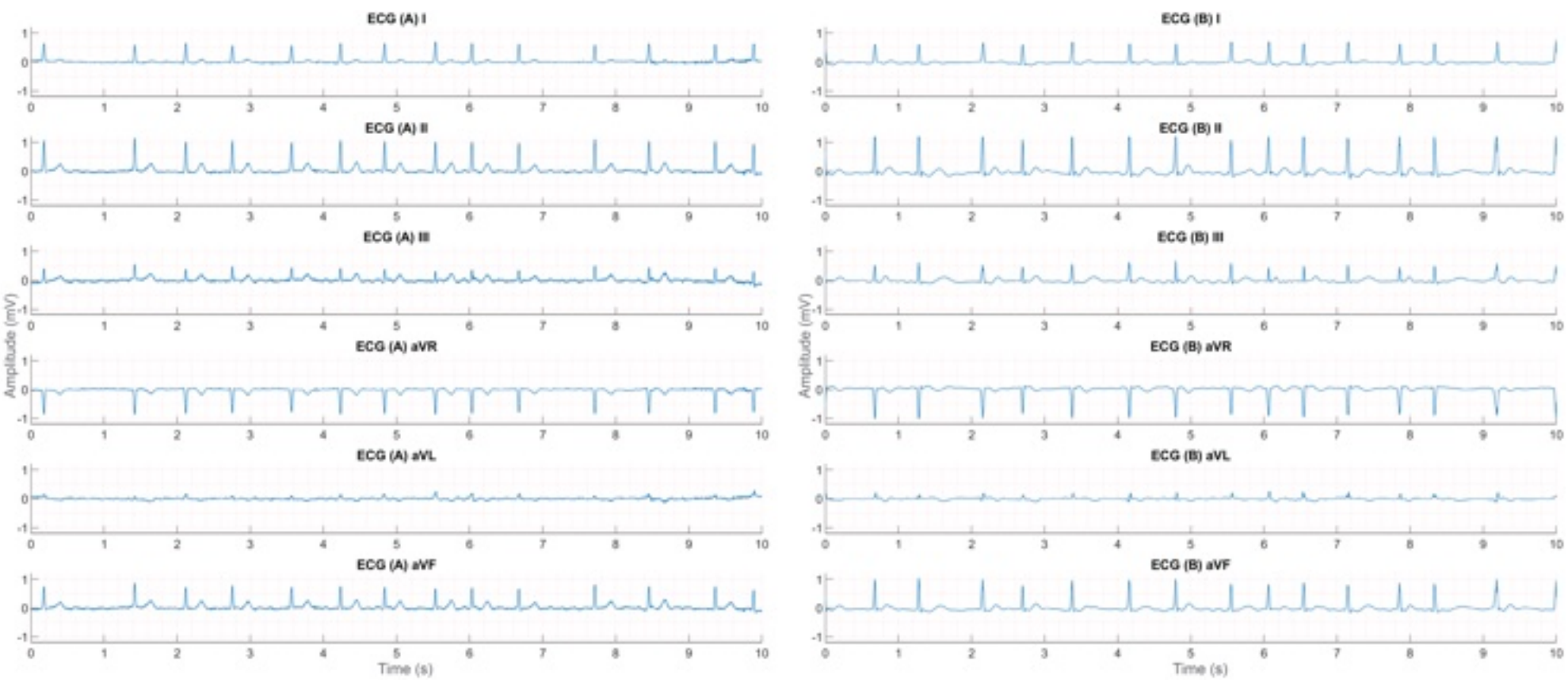

Fig. S6: Case 5. Atrial fibrillation

Case 5. Conventional ECG (A) vs Smartwatch (B)

Parameter	ECG A	ECG B	Difference (A-B)
Heart Rate (bpm)	86	93	-7
PR interval (ms)	-	-	-
QRS duration (ms)	91	97	-6
QT interval (ms)	354	370	-16
QTc (ms)	401	425	-24
RR interval (ms)	693	642	51
P wave duration (ms)	-	-	-
PP interval (ms)	-	-	-

Case 6

In panel A, sinus rhythm is present with a QRS showing left axis deviation, compatible with left anterior fascicular block (S wave in III > II, q wave in I and aVL). A subtle J-point elevation in I < 1 mm is consistent with lateral subendocardial ischemia. In panel B, the ischemic changes are harder to appreciate due to baseline oscillations, but the overall diagnosis remains the same.

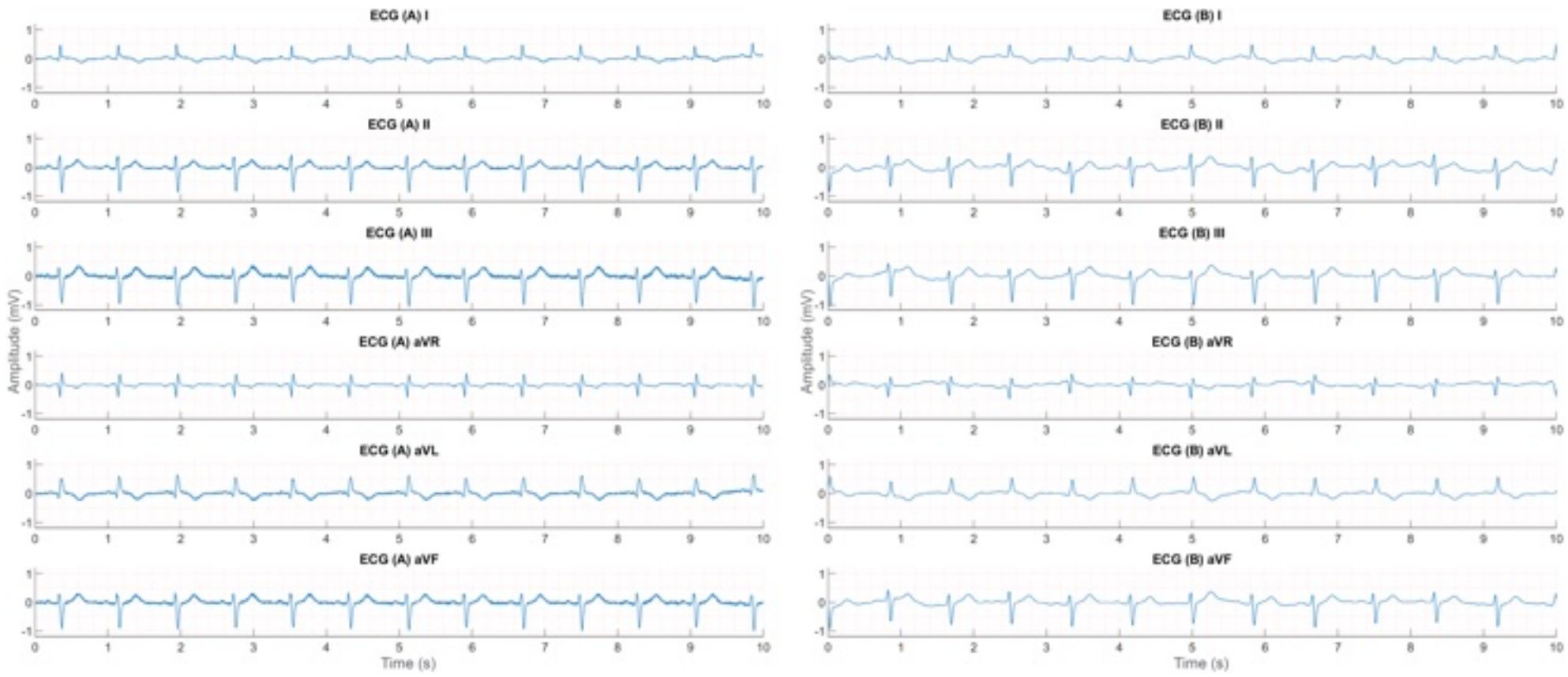

Fig. S7: Case 6. RBBB and LAFB with lateral ischemia

Case 6. Conventional ECG (A) vs Smartwatch (B)

Parameter	ECG A	ECG B	Difference (A-B)
Heart Rate (bpm)	75	72	3
PR interval (ms)	157	178	-21
QRS duration (ms)	105	116	-11
QT interval (ms)	404	434	-30
QTc (ms)	436	460	-24
RR interval (ms)	792	832	-40
P wave duration (ms)	95	122	-27
PP interval (ms)	792	832	-40

Case 7

In panel A, sinus rhythm is observed with a pathological Q wave in aVL and abnormal ventricular repolarization: slight ST-segment elevation in leads I and aVL with reciprocal ST depression in inferior leads (II, III, aVF). This is consistent with lateral necrosis and subendocardial ischemia. In panel B, QRS complexes and ST segments remain largely similar, allowing the same diagnosis despite minor differences in interval measurements.

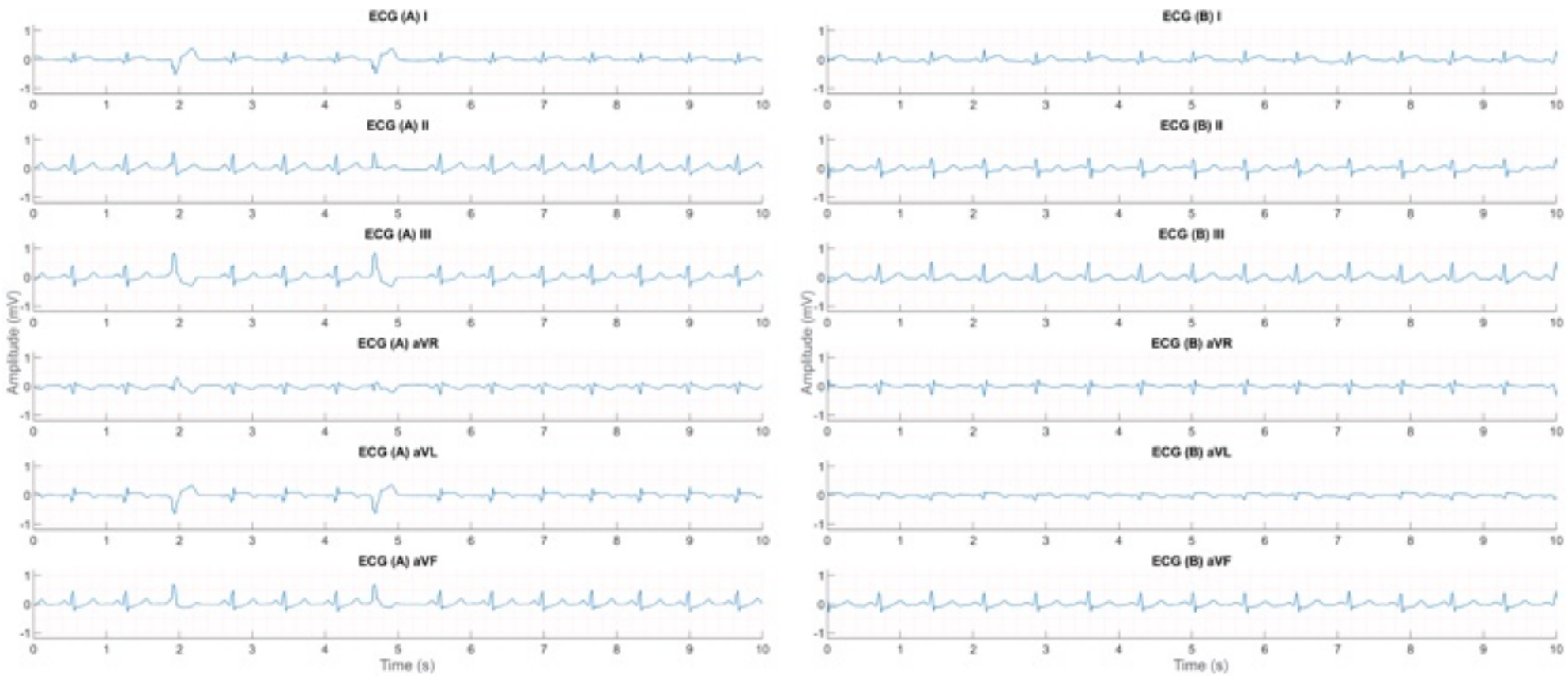

Fig. S8: Case 7. Anterolateral ischemia

Case 7. Conventional ECG (A) vs Smartwatch (B)

**Parameter**	**ECG A**	**ECG B**	**Difference (A-B)**
Heart Rate (bpm)	87	83	4
PR interval (ms)	156	159	-3
QRS duration (ms)	92	96	-4
QT interval (ms)	416	420	-4
QTc (ms)	465	463	2
RR interval (ms)	686	714	-28
P wave duration (ms)	117	126	-9
PP interval (ms)	686	714	-28

Case 8

In panel A, a sinus P wave is evident, followed by a wide QRS (>120 ms) with broad S waves in I and aVL and a QR morphology in aVR, consistent with complete right bundle branch block. In panel B, the P wave is less clear, and baseline oscillations in I, aVR, and aVL may suggest atrial fibrillation. However, clear P waves in II, III, and aVF, together with largely identical QRS morphology despite minor differences in interval measurements, confirm sinus rhythm with complete RBBB.

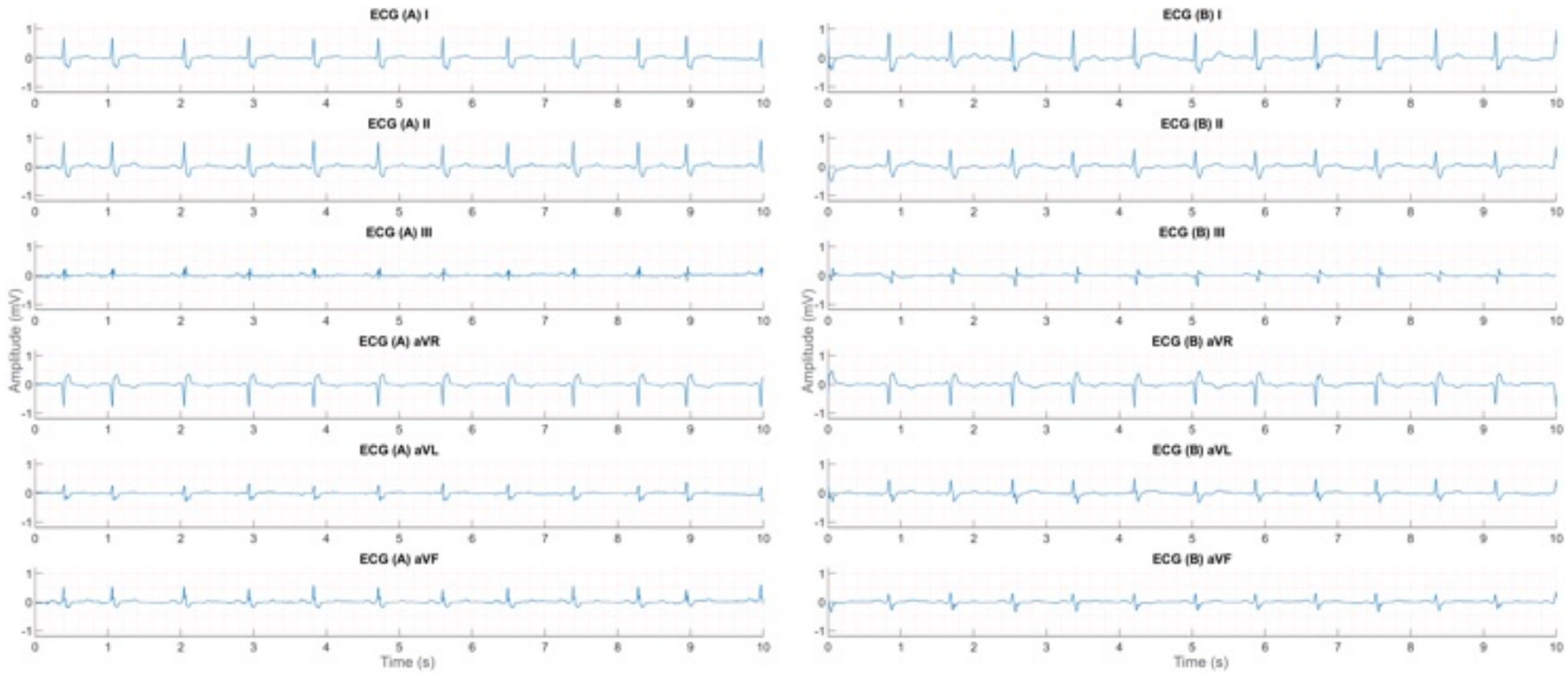

Fig. S9: Case 8. Complete right bundle branch block

Case 8. Conventional ECG (A) vs Smartwatch (B)

Parameter	ECG A	ECG B	Difference (A-B)
Heart Rate (bpm)	67	72	-5
PR interval (ms)	167	176	-9
QRS duration (ms)	132	135	-3
QT interval (ms)	441	431	10
QTc (ms)	458	457	1
RR interval (ms)	890	831	59
P wave duration (ms)	113	119	-6
PP interval (ms)	890	831	59

Case 9
In panel A, low-voltage atrial activity with P waves mainly in inferior leads (II, III, aVF) is seen, together with QRS complexes preceded by pacing spikes, consistent with a VDD pacing mode. In panel B, P waves are not visible, and pacing spikes are absent or of reduced amplitude, complicating recognition of a paced ventricular rhythm. Ventricular repolarization abnormalities are equally present in both tracings.

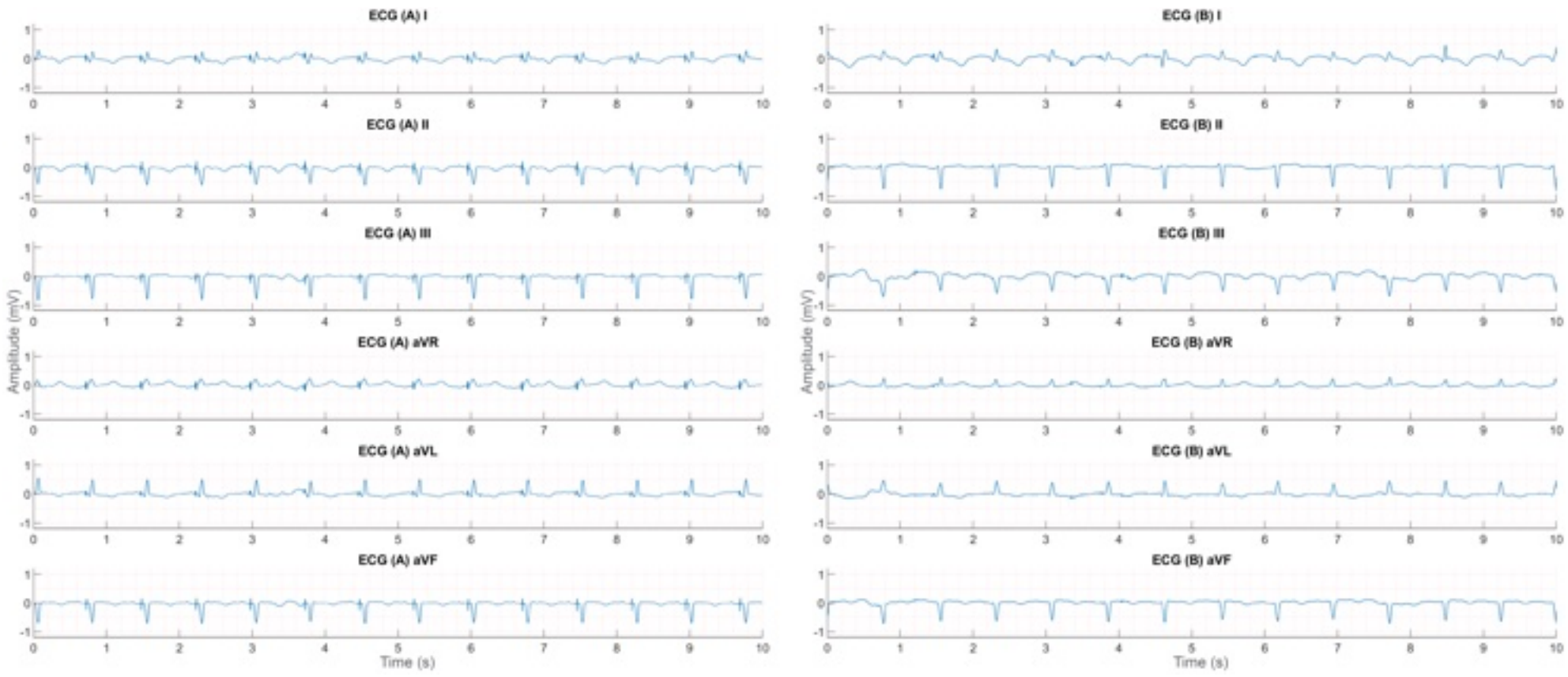

Fig. S10: Case 9. Ventricular paced rhythm (VDD)

Case 9. Conventional ECG (A) vs Smartwatch (B)

**Parameter**	**ECG A**	**ECG B**	**Difference (A-B)**
Heart Rate (bpm)	80	78	2
PR interval (ms)	150	NA	-
QRS duration (ms)	98	110	-12
QT interval (ms)	470	480	-10
QTc (ms)	509	515	-6
RR interval (ms)	748	768	-20
P wave duration (ms)	112	NA	-
PP interval (ms)	748	NA	-

NA: not assessable due to inability to reliably identify the waveform.

Case 10

In panel A, atrial pacing is clearly seen, with spikes preceding atrial activity across all leads. In panel B, pacing spikes are not visible, making it impossible to diagnose atrial pacing. Negative T waves in the inferior leads appear in both panels.

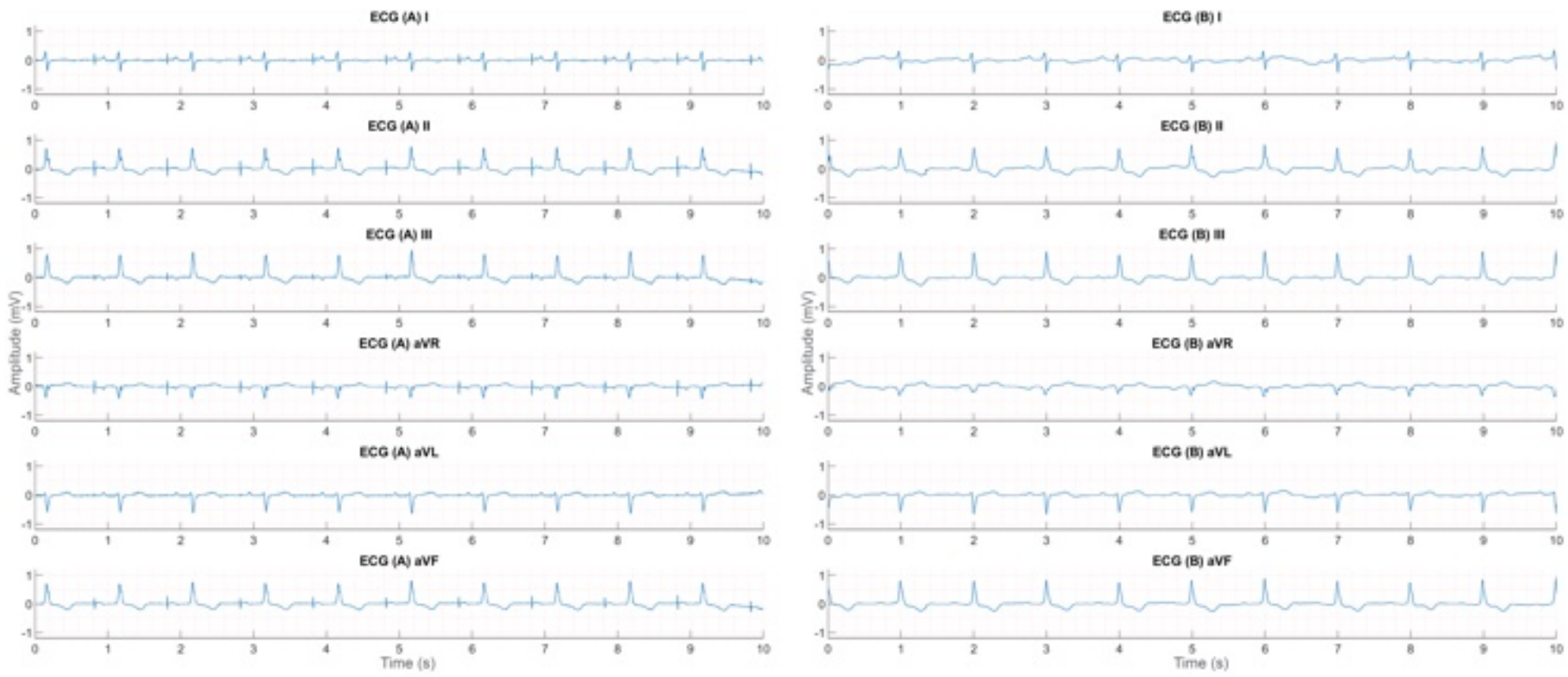

Fig. S11: Case 10. Atrial paced rhythm

Case 10. Conventional ECG (A) vs Smartwatch (B)

**Parameter**	**ECG A**	**ECG B**	**Difference (A-B)**
Heart Rate (bpm)	59	60	-1
PR interval (ms)	233	223	10
QRS duration (ms)	106	111	-5
QT interval (ms)	445	451	-6
QTc (ms)	445	451	-6
RR interval (ms)	1000	997	3
P wave duration (ms)	110	125	-15
PP interval (ms)	1000	997	3

Case 11
In panel A, sinus rhythm is present with left axis deviation fulfilling criteria for LAFB (S in III > II, q in I and aVL). Negative T waves are seen in I, especially in aVL. In panel B, baseline oscillations hinder the identification of the P wave and subtle ventricular repolarization abnormalities, with moderate to marked differences in interval measurements, particularly in QT, QTc, and P wave duration.

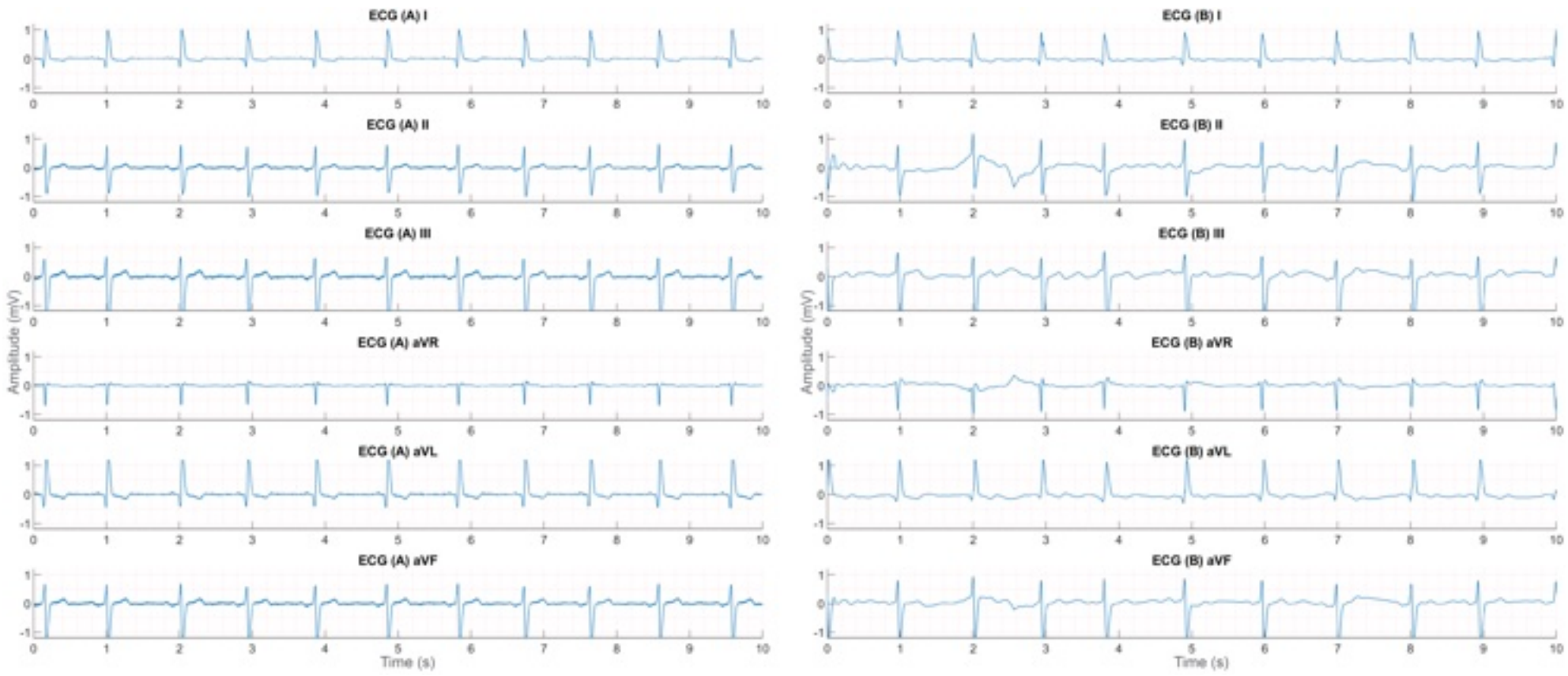

Fig. S12: Case 11. Sinus rhythm with LAFB and lateral T-wave inversion

Case 11. Conventional ECG (A) vs Smartwatch (B)

Parameter	ECG A	ECG B	Difference (A-B)
Heart Rate (bpm)	64	60	4
PR interval (ms)	171	170	1
QRS duration (ms)	125	140	-15
QT interval (ms)	404	448	-44
QTc (ms)	415	448	-33
RR interval (ms)	929	995	-66
P wave duration (ms)	88	123	-35
PP interval (ms)	929	995	-66

Case 12

In panel A, a wide-complex regular tachycardia is seen. On the right side of the tracing, a narrower beat compatible with a fusion complex confirms the diagnosis of ventricular tachycardia. Panel B shows identical QRS morphology, supporting the same diagnosis.

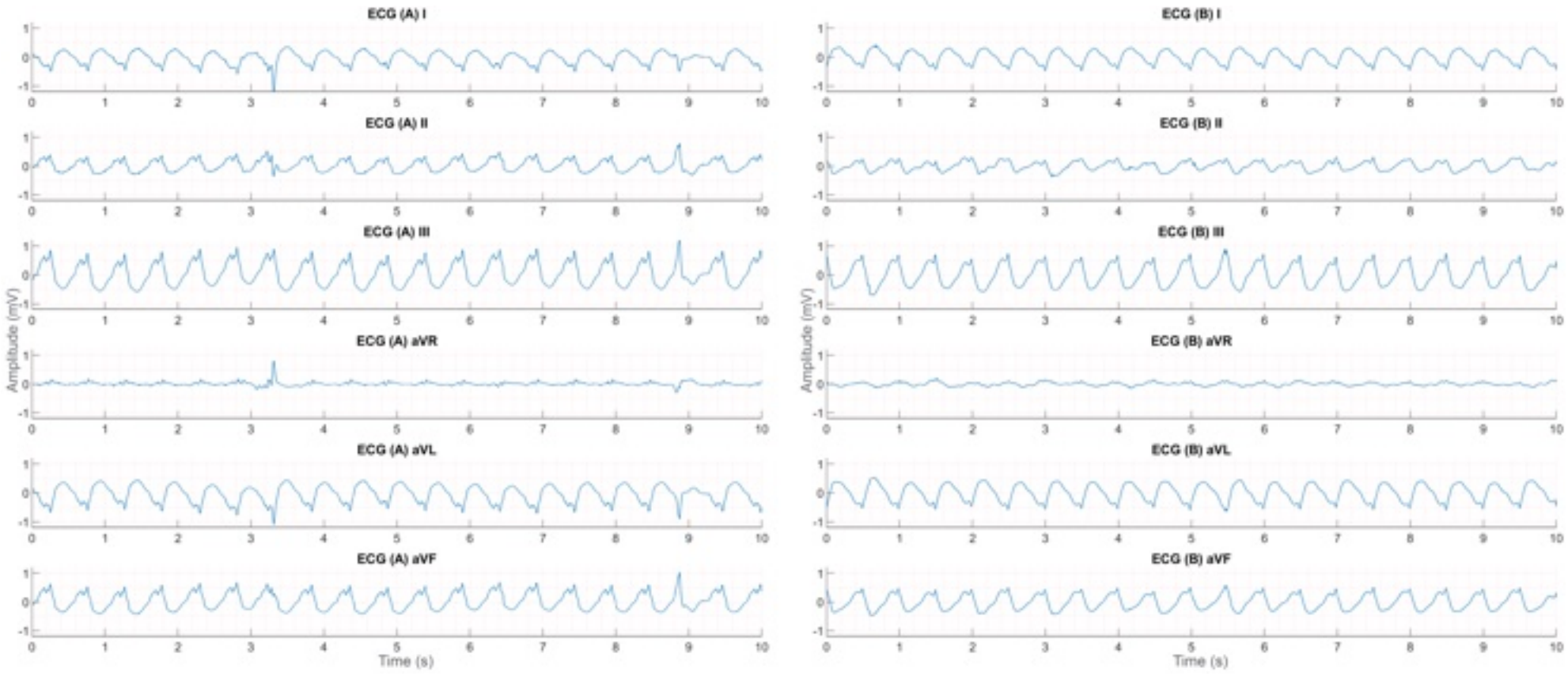

Fig. S13: Case 12. Ventricular tachycardia

Case 12. Conventional ECG (A) vs Smartwatch (B)

Parameter	ECG A	ECG B	Difference (A-B)
Heart Rate (bpm)	117	119	-2
PR interval (ms)	-	-	-
QRS duration (ms)	141	140	1
QT interval (ms)	403	434	-31
QTc (ms)	478	511	-33
RR interval (ms)	509	501	8
P wave duration (ms)	-	-	-
PP interval (ms)	-	-	-

Case 13

In panel A, sinus rhythm is present with QS complexes in III and aVF and ST-segment elevation in inferior leads with reciprocal ST depression in I and aVL, diagnostic of inferior STEMI. In panel B, the P wave is blunted, but QRS morphology and ST elevation remain evident.

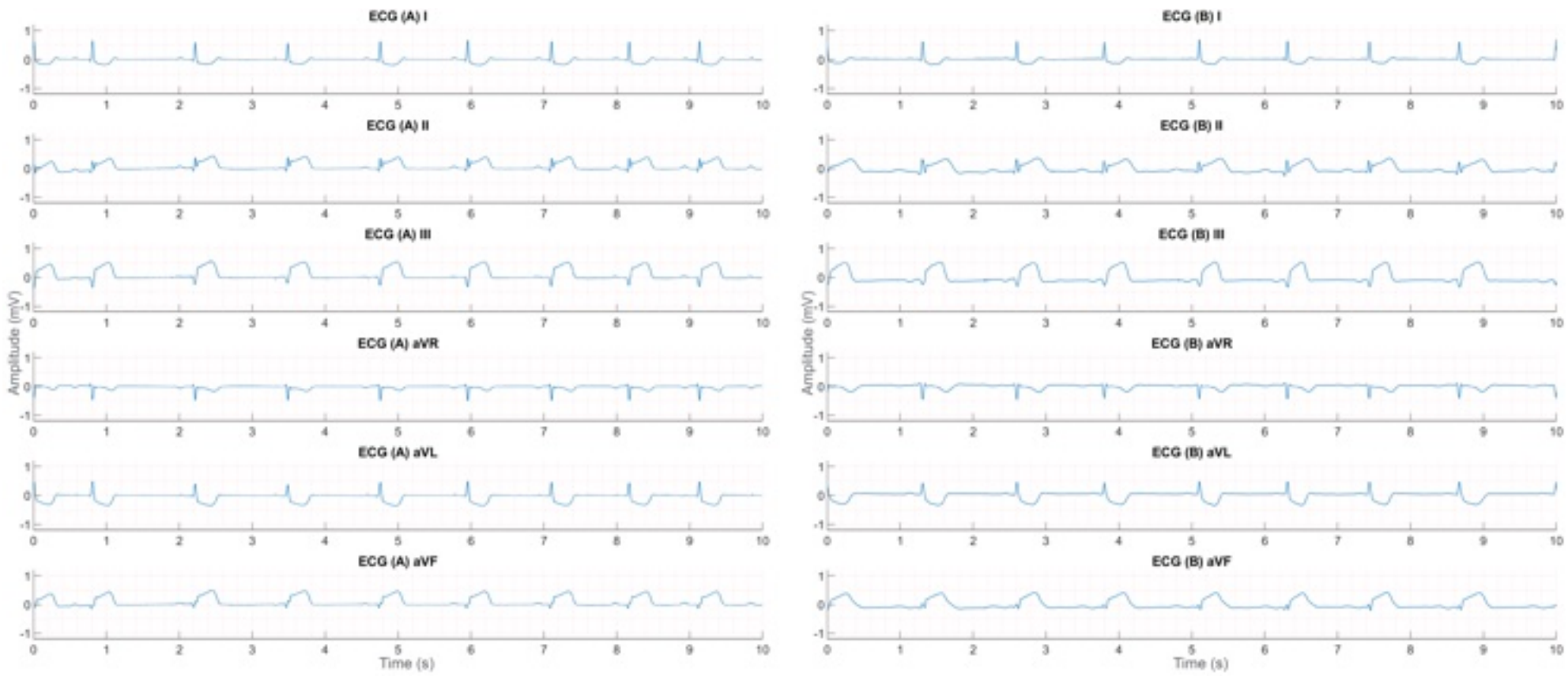

Fig. S14: Case 13. Inferior STEMI

Case 13. Conventional ECG (A) vs Smartwatch (B)

Parameter	ECG A	ECG B	Difference (A-B)
Heart Rate (bpm)	49	49	0
PR interval (ms)	199	175	24
QRS duration (ms)	101	118	-17
QT interval (ms)	422	477	-55
QTc (ms)	390	445	-55
RR interval (ms)	1208	1212	-4
P wave duration (ms)	112	119	-7
PP interval (ms)	1208	1212	-4

Case 14
In panel A, a DDD pacing mode is evident, with atrial and ventricular pacing spikes. In panel B, atrial spikes and P waves are not visible, and baseline oscillations (especially in lead II) could suggest atrial fibrillation erroneously.

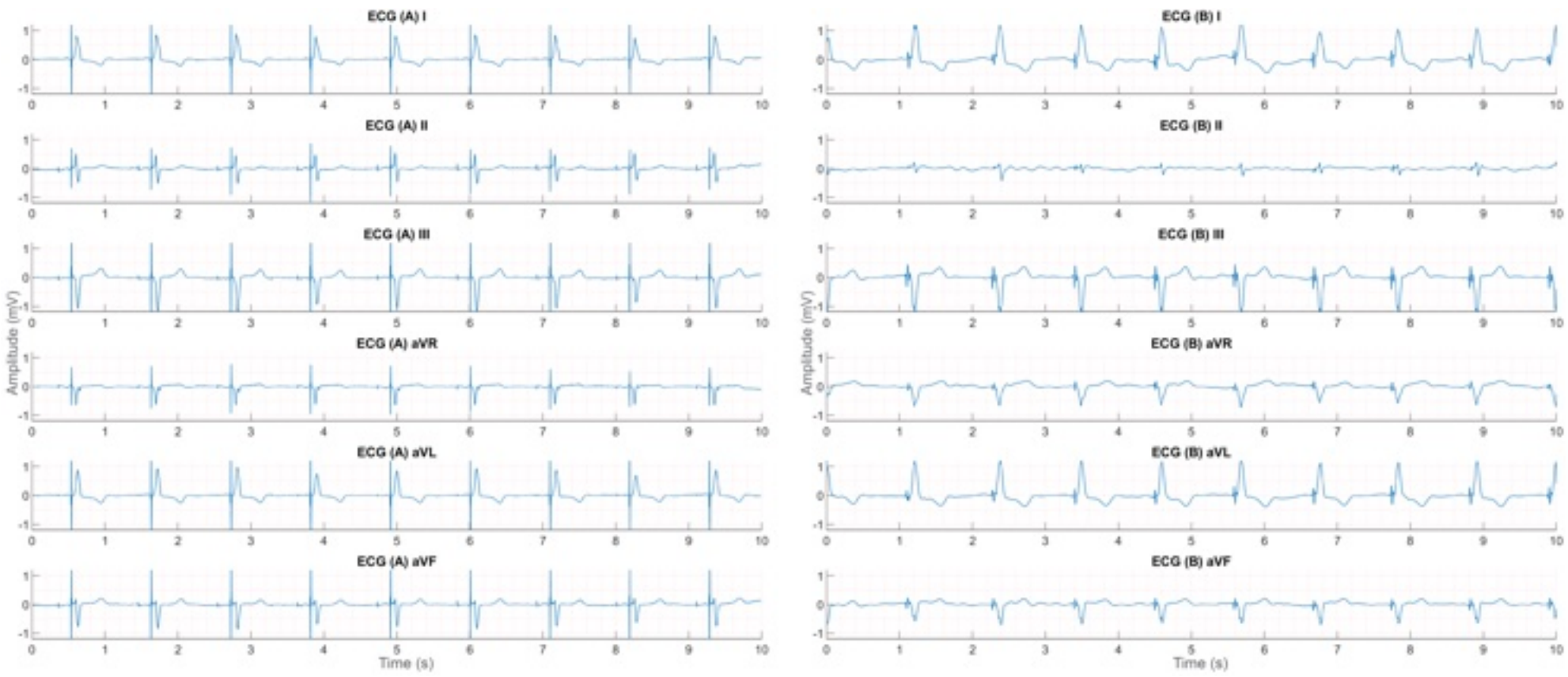

Fig. S15: Case 14. Dual-chamber paced rhythm (DDD)

Case 14. Conventional ECG (A) vs Smartwatch (B)

Parameter	ECG A	ECG B	Difference (A-B)
Heart Rate (bpm)	54	54	0
PR interval (ms)	151	159	-8
QRS duration (ms)	133	180	-47
QT interval (ms)	474	517	-43
QTc (ms)	459	502	-43
RR interval (ms)	1093	1100	-7
P wave duration (ms)	100	82	18
PP interval (ms)	1093	1100	-7

Case 15

In panel A, a sinus rhythm at 100 bpm is observed. QRS morphology is normal, but ventricular repolarization is abnormal with negative T waves in inferior leads (II, III, aVF), consistent with inferior subendocardial ischemia. In panel B, the P wave is less clear, though still visible in I and II. QRS and T-wave morphology are similar, but with slightly lower amplitude.

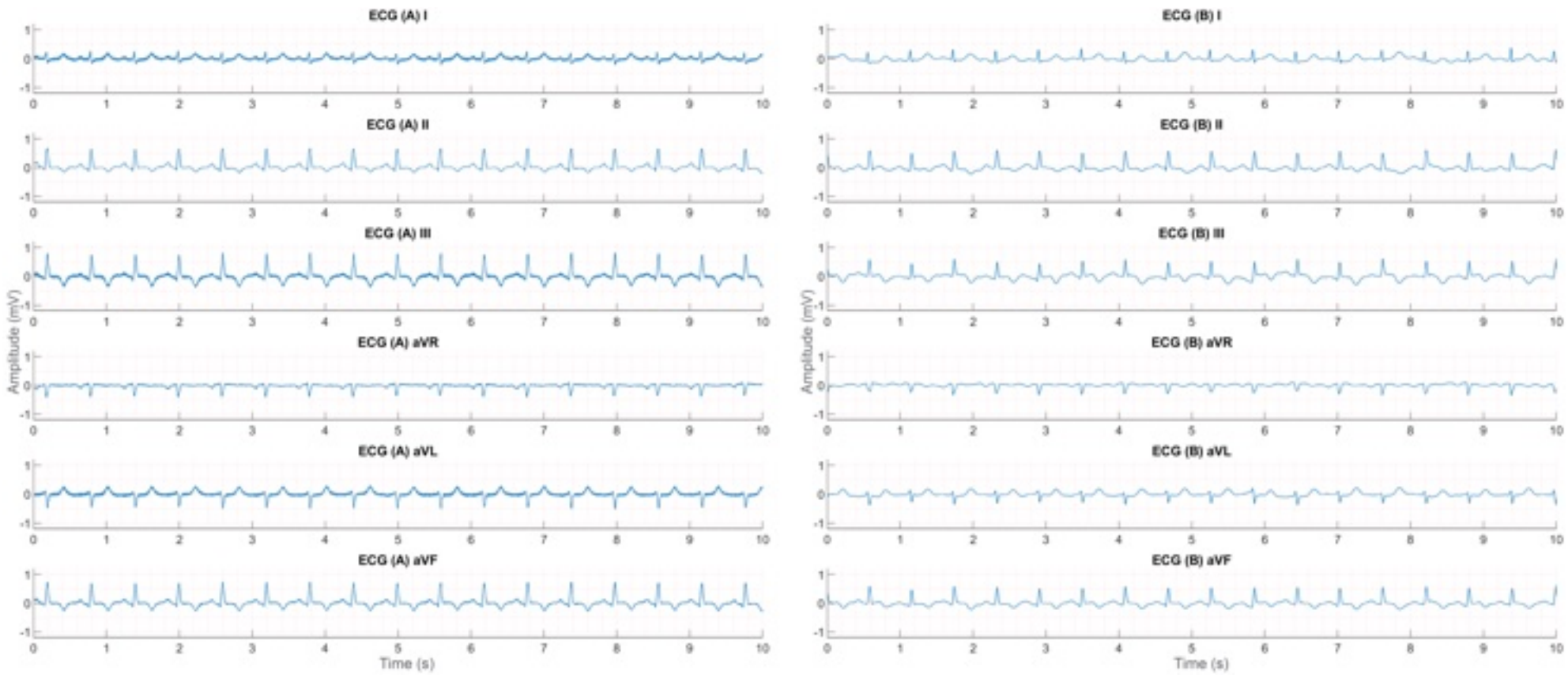

Fig. S16: Case 15. Inferior ischemia

Case 15. Conventional ECG (A) vs Smartwatch (B)

Parameter	ECG A	ECG B	Difference (A-B)
Heart Rate (bpm)	100	101	-1
PR interval (ms)	162	178	-16
QRS duration (ms)	91	87	4
QT interval (ms)	374	381	-7
QTc (ms)	435	444	-9
RR interval (ms)	598	590	8
P wave duration (ms)	102	122	-20
PP interval (ms)	598	590	8

Case 16
In panel A, sinus rhythm is present with a broad QRS (144 ms), predominantly positive in I and aVL, consistent with complete left bundle branch block. Secondary repolarization abnormalities are evident. In panel B, P-wave, QRS, and repolarization morphology are comparable.

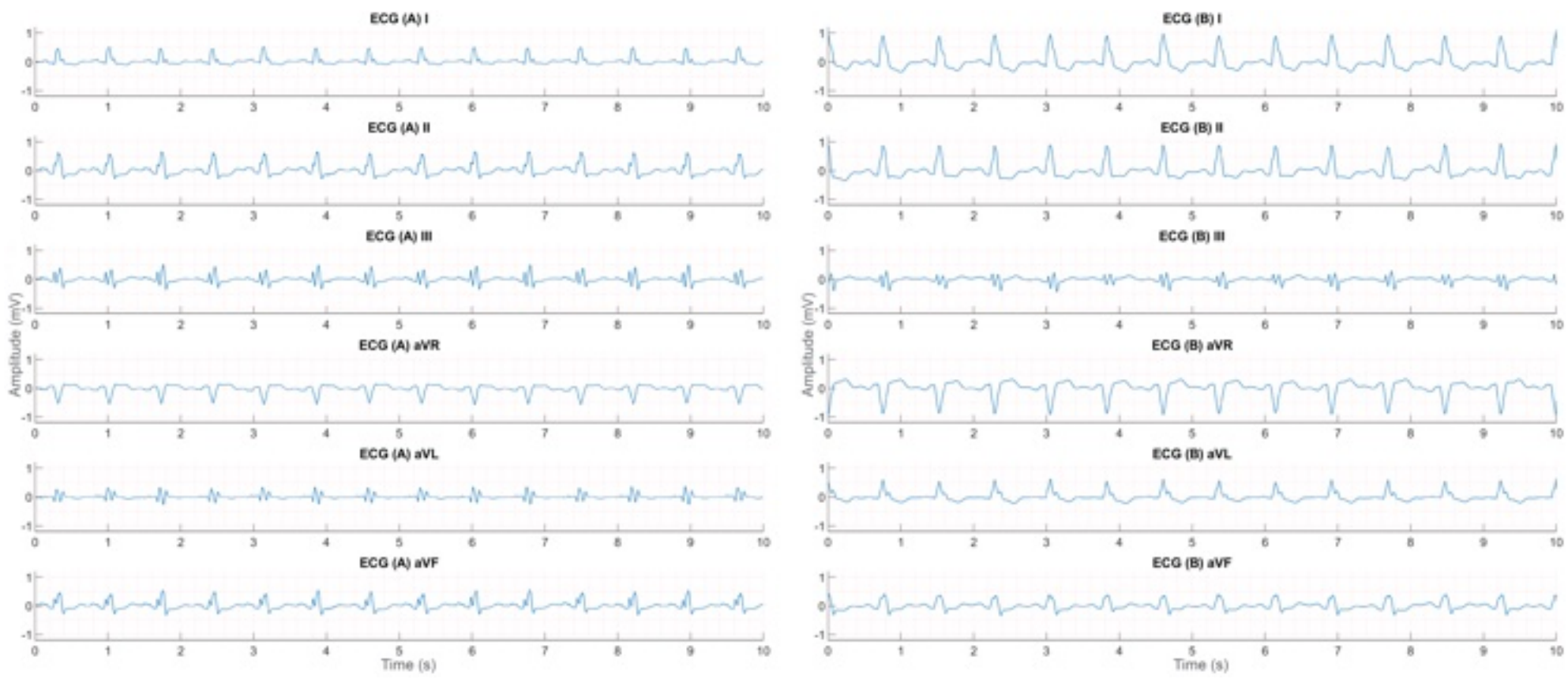

Fig. S17: Case 16. Left bundle branch block

Case 16. Conventional ECG (A) vs Smartwatch (B)

Parameter	ECG A	ECG B	Difference (A-B)
Heart Rate (bpm)	82	77	5
PR interval (ms)	178	175	3
QRS duration (ms)	144	153	-9
QT interval (ms)	443	454	-11
QTc (ms)	485	489	-4
RR interval (ms)	724	771	-47
P wave duration (ms)	124	131	-7
PP interval (ms)	724	771	-47

Case 17

In panel A, no P waves are visible. Very rapid low-amplitude atrial activity (230 bpm), best seen in inferior leads, suggests atypical atrial flutter or organized atrial fibrillation. QRS intervals are narrow with non-pathological Q waves inferiorly. In panel B, atrial activity is absent, suggesting atrial fibrillation. QRS and repolarization morphology remain similar.

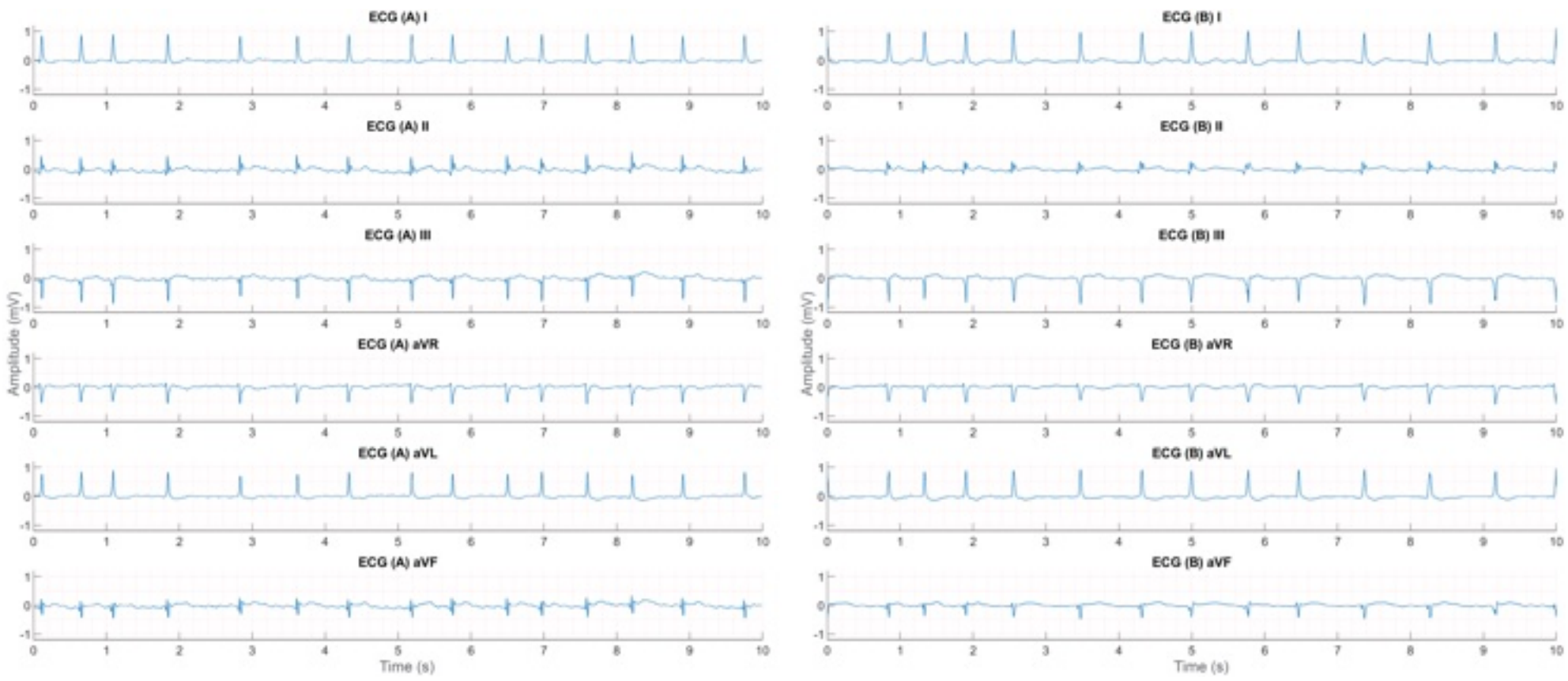

Fig. S18: Case 17. Atrial fibrillation

Case 17. Conventional ECG (A) vs Smartwatch (B)

Parameter	ECG A	ECG B	Difference (A-B)
Heart Rate (bpm)	87	77	10
PR interval (ms)	-	-	-
QRS duration (ms)	92	106	-14
QT interval (ms)	358	412	-54
QTc (ms)	406	446	-40
RR interval (ms)	687	779	-92
P wave duration (ms)	-	-	-
PP interval (ms)	-	-	-

Case 18

In panel A, sinus P waves at 120 bpm are seen with progressive PR prolongation (>200 ms) until a non-conducted P wave, consistent with second-degree AV block (Mobitz I). QRS morphology is widened with left axis deviation and Q waves in I and aVL. ST elevation in I and aVL with reciprocal depression inferiorly suggests lateral subendocardial ischemia. In panel B, P waves are absent in most leads but occasionally visible in II, and ST elevation remains apparent.

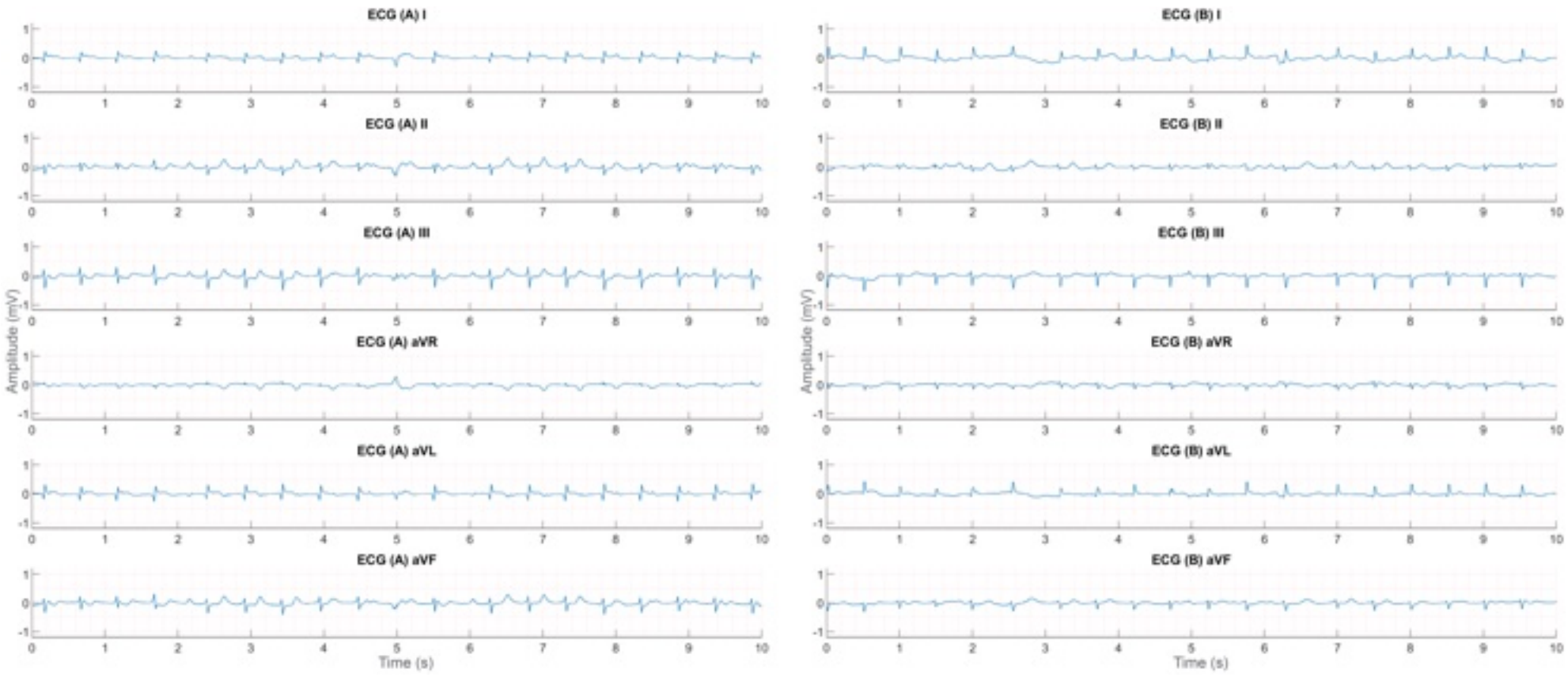

Fig. S19: Case 18. Sinus tachycardia with AV block (Mobitz I) and lateral ischemia

Case 18. Conventional ECG (A) vs Smartwatch (B)

Parameter	ECG A	ECG B	Difference (A-B)
Heart Rate (bpm)	116	117	-1
PR interval (ms)	-	-	-
QRS duration (ms)	88	97	-9
QT interval (ms)	349	358	-9
QTc (ms)	424	409	15
RR interval (ms)	514	512	2
P wave duration (ms)	104	108	-4
PP interval (ms)	-	-	-

Case 19
In panel A, an atypical atrial flutter is observed with positive F waves in inferior leads and left-axis QRS morphology (positive in I, S > R in aVF). In panel B, sinusoidal atrial activity and regular ventricular response suggest atrial flutter. QRS morphology is unchanged.

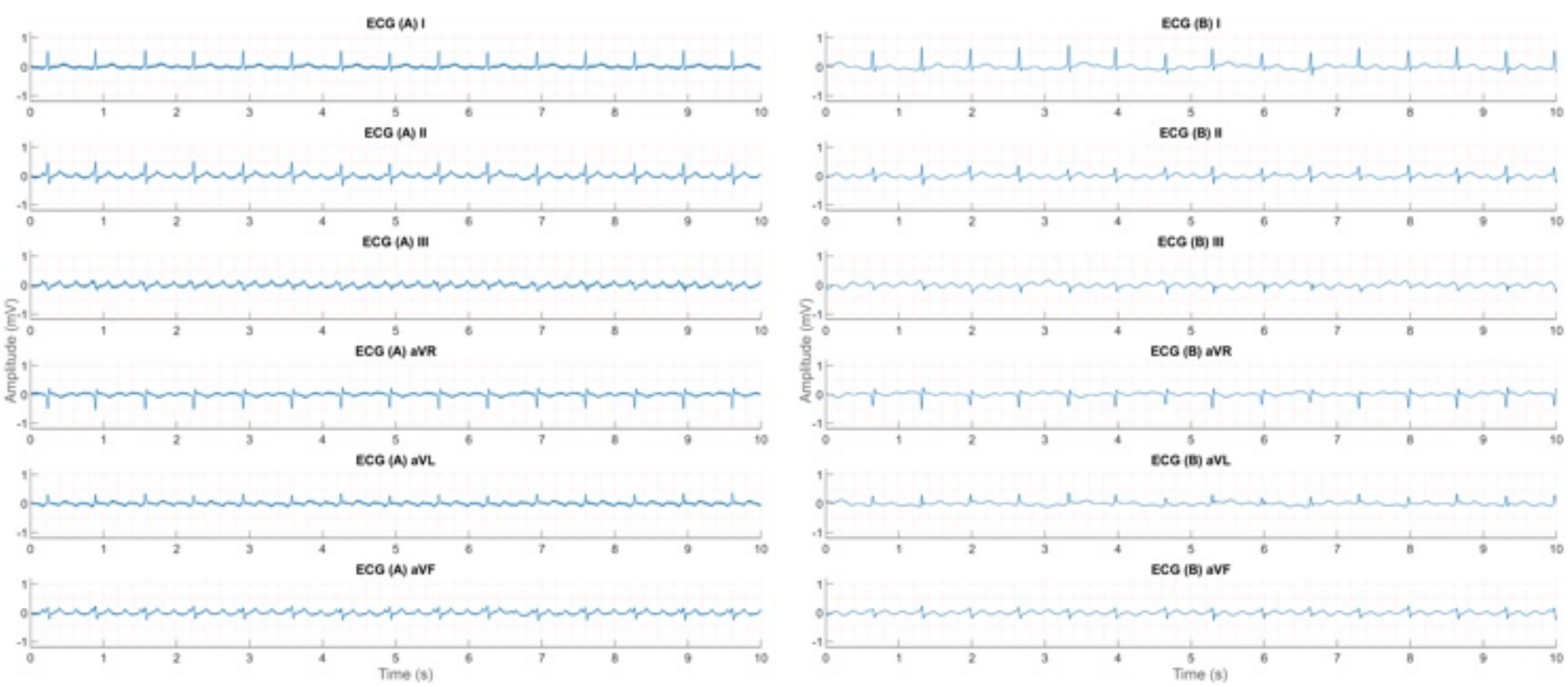

Fig. S20: Case 19. Atypical atrial flutter

Case 19. Conventional ECG (A) vs Smartwatch (B)

Parameter	ECG A	ECG B	Difference (A-B)
Heart Rate (bpm)	89	89	0
PR interval (ms)	-	-	-
QRS duration (ms)	82	97	-15
QT interval (ms)	343	358	-15
QTc (ms)	394	409	-15
RR interval (ms)	668	669	-1
P wave duration (ms)	-	-	-
PP interval (ms)	-	-	-

Case 20
In panel A, typical atrial flutter is seen with characteristic sawtooth F waves in inferior leads. In panel B, F waves are not visible, and baseline oscillations could suggest atrial fibrillation, but QRS morphology remains identical.

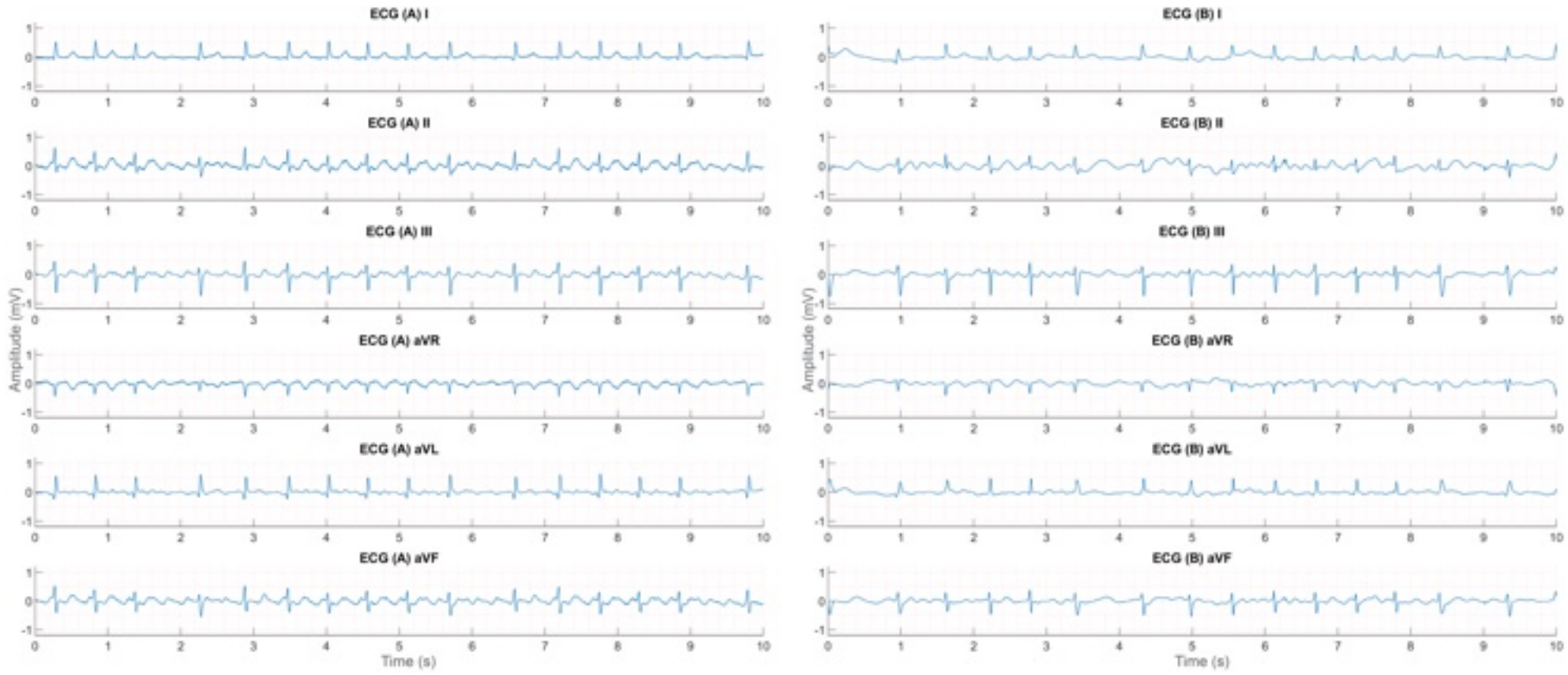

Fig. S21: Case 20. Typical atrial flutter

Case 20. Conventional ECG (A) vs Smartwatch (B)

**Parameter**	**ECG A**	**ECG B**	**Difference (A-B)**
Heart Rate (bpm)	105	96	9
PR interval (ms)	-	-	-
QRS duration (ms)	94	99	-5
QT interval (ms)	391	391	0
QTc (ms)	457	449	8
RR interval (ms)	569	619	-50
P wave duration (ms)	-	-	-
PP interval (ms)	-	-	-

Non-standard Acronyms and Abbreviations (Tables)

SP: Specialty
ADx: Accurate Diagnosis
AxC: Axis Concordance
SDB: Same Diagnosis in Both
CS: Correlation Score
AIES: Accuracy Identifying ECG Source
YoE: Years of Experience

Table S1: Case 1. Complete AV block

SP	ADx [n (%)]	AxC Yes [n (%)]	SDB Yes [n (%)]	CS (Mean ± SD)	AIES [n (%)]
Cardiology	48 (96%)	50 (100%)	48 (96%)	86.8 ± 14.3	41 (82%)
Nurse	14 (93.3%)	15 (100%)	13 (86.6%)	80.4 ± 15.7	14 (93.3%)
G. Practitioner	7 (70%)	10 (100%)	9 (90%)	82 ± 22.6	9 (90%)
I. Medicine	3 (60%)	4 (80%)	5 (100%)	89 ± 10.2	4 (80%)
E. Department	3 (60%)	5 (100%)	5 (100%)	100 ± 0	4 (80%)
**Total**	**75 (88.2%)**	**84 (98.8%)**	**80 (94.1%)**	**86 ± 14.9**	**72 (84.7%)**

Table S2: Case 2. SR

SP	ADx [n (%)]	AxC Yes [n (%)]	SDB Yes [n (%)]	CS (Mean ± SD)	AIES [n (%)]
Cardiology	47 (94%)	50 (100%)	50 (100%)	96.6 ± 5	35 (70%)
Nurse	15 (100%)	14 (93.3%)	15 (100%)	94.8 ± 6.9	14 (93.3%)
G. Practitioner	9 (90%)	9 (90%)	10 (100%)	95 ± 6.6	7 (70%)
I. Medicine	5 (100%)	5 (100%)	5 (100%)	97.8 ± 4.3	3 (60%)
E. Department	5 (100%)	5 (100%)	5 (100%)	100 ± 0	5 (100%)
**Total**	**81 (95.2%)**	**83 (97.6%)**	**85 (100%)**	**96.3 ± 5.4**	**64 (75.2%)**

Table S3: Case 3. First degree AV block

SP	ADx [n (%)]	AxC Yes [n (%)]	SDB Yes [n (%)]	CS (Mean ± SD)	AIES [n (%)]
Cardiology	45 (90%)	49 (98%)	47 (94%)	92 ± 11.1	39 (78%)
Nurse	13 (86.6%)	13 (86.6%)	14 (93.3%)	85.6 ± 13.3	11 (73.3%)
G. Practitioner	10 (100%)	10 (100%)	10 (100%)	90 ± 14.3	4 (40%)
I. Medicine	4 (80%)	5 (100%)	4 (80%)	93.8 ± 13.3	2 (40%)
E. Department	5 (100%)	5 (100%)	5 (100%)	98 ± 4.4	5 (100%)
**Total**	**77 (90.5%)**	**82 (96.4%)**	**80 (94.1%)**	**91.1 ± 11.9**	**61 (71.7%)**

Table S4: Case 4. 2:1 AV block with RBBB

SP	ADx [n (%)]	AxC Yes [n (%)]	SDB Yes [n (%)]	CS (Mean ± SD)	AIES [n (%)]
Cardiology	40 (80%)	50 (100%)	40 (80%)	77.6 ± 18.4	31 (62%)
Nurse	8 (53.3%)	12 (80%)	8 (53.3%)	70.2 ± 23.3	6 (40%)
G. Practitioner	4 (40%)	10 (100%)	10 (100%)	87 ± 12.7	3 (30%)
I. Medicine	2 (40%)	4 (80%)	5 (100%)	85.6 ± 16.3	2 (40%)
E. Department	2 (40%)	5 (100%)	5 (100%)	95 ± 11.1	4 (80%)
**Total**	**56 (65.9%)**	**81 (95.2%)**	**68 (80%)**	**78.9 ± 19.1**	**46 (54.1%)**

Table S5: Case 5. AF

SP	ADx [n (%)]	AxC Yes [n (%)]	SDB Yes [n (%)]	CS (Mean ± SD)	AIES [n (%)]
Cardiology	49 (98%)	50 (100%)	50 (100%)	94.5 ± 6.1	25 (50%)
Nurse	15 (100%)	13 (86.6%)	15 (100%)	91.3 ± 11.8	7 (46.6%)
G. Practitioner	8 (80%)	10 (100%)	9 (90%)	86.5 ± 17.9	5 (50%)
I. Medicine	4 (80%)	5 (100%)	5 (100%)	99 ± 2.2	1 (20%)
E. Department	5 (100%)	5 (100%)	5 (100%)	100 ± 0	4 (80%)
**Total**	**81 (95.2%)**	**83 (97.6%)**	**84 (98.8%)**	**93.6 ± 9.5**	**42 (49.4%)**

Table S6: Case 6. RBBB and LAFB. Lateral ischemia

SP	ADx [n (%)]	AxC Yes [n (%)]	SDB Yes [n (%)]	CS (Mean ± SD)	AIES [n (%)]
Cardiology	39 (78%)	50 (100%)	48 (96%)	91.2 ± 9.2	34 (68%)
Nurse	15 (100%)	14 (93.3%)	14 (93.3%)	86.3 ± 13.6	12 (80%)
G. Practitioner	7 (70%)	10 (100%)	9 (90%)	85.5 ± 17.2	7 (70%)
I. Medicine	5 (100%)	5 (100%)	5 (100%)	98.8 ± 1.9	2 (40%)
E. Department	5 (100%)	5 (100%)	5 (100%)	100 ± 0	5 (100%)
**Total**	**79 (93%)**	**84 (98%)**	**81 (95.2%)**	**90.6 ± 11.3**	**60 (70.5%)**

Table S7: Case 7. Anterolateral ischemia

SP	ADx [n (%)]	AxC Yes [n (%)]	SDB Yes [n (%)]	CS (Mean ± SD)	AIES [n (%)]
Cardiology	42 (84%)	48 (94%)	42 (84%)	82.3 ± 17.8	38 (76%)
Nurse	9 (60%)	12 (80%)	10 (66.6%)	73.4 ± 25.3	13 (86.6%)
G. Practitioner	2 (20%)	9 (90%)	6 (60%)	63 ± 20.5	6 (60%)
I. Medicine	2 (40%)	5 (100%)	5 (100%)	94.8 ± 8.5	4 (80%)
E. Department	2 (40%)	5 (100%)	4 (80%)	76.2 ± 42.9	4 (80%)
**Total**	**57 (67%)**	**79 (92.9%)**	**67 (78.8%)**	**78.8 ± 22.2**	**65 (76.4%)**

Table S8: Case 8. RBBB

SP	ADx [n (%)]	AxC Yes [n (%)]	SDB Yes [n (%)]	CS (Mean ± SD)	AIES [n (%)]
Cardiology	42 (84%)	49 (98%)	47 (94%)	88.5 ± 12.5	42 (84%)
Nurse	8 (53.3%)	12 (80%)	11 (73.3%)	81.2 ± 12.8	11 (73.3%)
G. Practitioner	5 (50%)	10 (100%)	9 (100%)	88 ± 20.9	3 (30%)
I. Medicine	2 (40%)	4 (80%)	4 (80%)	80.8 ± 39.6	4 (80%)
E. Department	4 (80%)	4 (80%)	5 (100%)	99 ± 2.2	4 (80%)
**Total**	**61 (71.8%)**	**79 (92.9%)**	**76 (89.4%)**	**87.3 ± 16**	**64 (75.2%)**

Table S9: Case 9. Paced ventricular rhythm

SP	ADx [n (%)]	AxC Yes [n (%)]	SDB Yes [n (%)]	CS (Mean ± SD)	AIES [n (%)]
Cardiology	50 (100%)	50 (100%)	20 (40%)	53.3 ± 24.2	49 (98%)
Nurse	14 (93.3%)	9 (60%)	2 (13.3%)	47.7 ± 24.7	15 (100%)
G. Practitioner	7 (70%)	10 (100%)	2 (20%)	49.1 ± 30.1	9 (90%)
I. Medicine	3 (60%)	4 (80%)	1 (20%)	50 ± 29.1	5 (100%)
E. Department	5 (100%)	5 (100%)	1 (20%)	43.3 ± 41.8	5 (100%)
**Total**	**79 (92.9%)**	**78 (91.7%)**	**26 (30.5%)**	**51 ± 26**	**83 (97.6%)**

Table S10: Case 10. Atrial rhythm

SP	ADx [n (%)]	AxC Yes [n (%)]	SDB Yes [n (%)]	CS (Mean ± SD)	AIES [n (%)]
Cardiology	48 (96%)	48 (96%)	10 (20%)	47.1 ± 26.7	49 (98%)
Nurse	13 (86.6%)	14 (93.3%)	2 (13.3%)	52 ± 23.6	14 (93.3%)
G. Practitioner	7 (70%)	10 (100%)	2 (20%)	39.1 ± 25.7	8 (80%)
I. Medicine	4 (80%)	4 (80%)	1 (20%)	54 ± 32	5 (100%)
E. Department	3 (60%)	5 (100%)	2 (40%)	40.4 ± 43.7	5 (100%)
**Total**	**75 (88.2%)**	**81 (95.2%)**	**17 (20%)**	**47 ± 27.1**	**81 (95.2%)**

Table S11: Case 11. SR, LAFB and lateral negative T-waves

SP	ADx [n (%)]	AxC Yes [n (%)]	SDB Yes [n (%)]	CS (Mean ± SD)	AIES [n (%)]
Cardiology	35 (70%)	49 (98%)	38 (76%)	70.5 ± 28.1	42 (84%)
Nurse	12 (80%)	14 (93.3%)	6 (40%)	58 ± 23.6	14 (93.3%)
G. Practitioner	5 (50%)	10 (100%)	3 (30%)	59 ± 31	5 (50%)
I. Medicine	2 (40%)	4 (80%)	3 (60%)	60 ± 33.9	4 (80%)
E. Department	5 (100%)	5 (100%)	4 (80%)	82 ± 30.3	4 (80%)
**Total**	**59 (69.4%)**	**82 (96.4%)**	**54 (63.5%)**	**67 ± 28.3**	**69 (81.1%)**

Table S12: Case 12. VT

SP	ADx [n (%)]	AxC Yes [n (%)]	SDB Yes [n (%)]	CS (Mean ± SD)	AIES [n (%)]
Cardiology	48 (96%)	50 (100%)	49 (98%)	92.5 ± 12.9	37 (74%)
Nurse	15 (100%)	15 (100%)	15 (100%)	96.4 ± 3.9	11 (73.3%)
G. Practitioner	10 (100%)	10 (100%)	10 (100%)	95 ± 5.2	7 (70%)
I. Medicine	5 (100%)	5 (100%)	5 (100%)	96.6 ± 6.5	4 (80%)
E. Department	4 (80%)	5 (100%)	5 (100%)	100 ± 0	5 (100%)
**Total**	**82 (96.4%)**	**85 (100%)**	**84 (98.8%)**	**94.2 ± 10.5**	**64 (75.2%)**

Table S13: Case 13. Inferior STEMI

SP	ADx [n (%)]	AxC Yes [n (%)]	SDB Yes [n (%)]	CS (Mean ± SD)	AIES [n (%)]
Cardiology	50 (100%)	50 (100%)	50 (100%)	96.6 ± 4.2	42 (84%)
Nurse	15 (100%)	15 (100%)	15 (100%)	88.4 ± 18.8	13 (86.6%)
G. Practitioner	8 (80%)	10 (100%)	10 (100%)	96.5 ± 4.1	5 (50%)
I. Medicine	5 (100%)	5 (100%)	5 (100%)	99.2 ± 1	2 (40%)
E. Department	4 (80%)	5 (100%)	5 (100%)	100 ± 0	5 (100%)
**Total**	**82 (96.4%)**	**85 (100%)**	**85 (100%)**	**95.5 ± 9.1**	**67 (78.8%)**

Table S14: Case 14. Paced AV Rhythm

SP	ADx [n (%)]	AxC Yes [n (%)]	SDB Yes [n (%)]	CS (Mean ± SD)	AIES [n (%)]
Cardiology	50 (100%)	49 (98%)	19 (38%)	62.8 ± 21.7	47 (94%)
Nurse	15 (100%)	14 (93.3%)	8 (53.3%)	76 ± 15.4	14 (93.3%)
G. Practitioner	10 (100%)	9 (90%)	5 (50%)	63.6 ± 34.2	6 (60%)
I. Medicine	5 (100%)	5 (100%)	3 (60%)	87 ± 10.3	4 (80%)
E. Department	4 (80%)	5 (100%)	4 (80%)	94 ± 13.4	4 (80%)
**Total**	**84 (98.7%)**	**82 (96.4%)**	**39 (45.8%)**	**68.4 ± 23.4**	**75 (88.2%)**

Table S15: Case 15. Inferior ischemia

SP	ADx [n (%)]	AxC Yes [n (%)]	SDB Yes [n (%)]	CS (Mean ± SD)	AIES [n (%)]
Cardiology	47 (94%)	50 (100%)	45 (90%)	83.4 ± 19.6	39 (78%)
Nurse	12 (80%)	14 (93.3%)	15 (100%)	86.6 ± 12.8	12 (80%)
G. Practitioner	9 (90%)	10 (100%)	9 (90%)	80 ± 17.7	5 (50%)
I. Medicine	2 (40%)	5 (100%)	4 (80%)	88 ± 8.3	5 (100%)
E. Department	4 (80%)	5 (100%)	5 (100%)	100 ± 0	5 (100%)
**Total**	**74 (87.1%)**	**84 (98.8%)**	**78 (91.7%)**	**84.8 ± 17.5**	**66 (77.6%)**

Table S16: Case 16. LBBB

SP	ADx [n (%)]	AxC Yes [n (%)]	SDB Yes [n (%)]	CS (Mean ± SD)	AIES [n (%)]
Cardiology	46 (92%)	47 (94%)	48 (96%)	87 ± 13.1	22 (44%)
Nurse	11 (73.3%)	12 (80%)	13 (86.6%)	77.2 ± 19.1	11 (73.3%)
G. Practitioner	5 (50%)	10 (100%)	10 (100%)	81 ± 15	5 (50%)
I. Medicine	0 (0%)	5 (100%)	4 (80%)	88 ± 8.3	5 (100%)
E. Department	5 (100%)	5 (100%)	5 (100%)	100 ± 0	4 (80%)
**Total**	**67 (78.8%)**	**79 (92.9%)**	**80 (94.1%)**	**84.9 ± 15.1**	**47 (55.2%)**

Table S17: Case 17. AF

SP	ADx [n (%)]	AxC Yes [n (%)]	SDB Yes [n (%)]	CS (Mean ± SD)	AIES [n (%)]
Cardiology	37 (74%)	50 (100%)	41 (82%)	83.1 ± 19.3	42 (84%)
Nurse	12 (80%)	13 (86.6%)	12 (80%)	86.4 ± 15.4	8 (53.3%)
G. Practitioner	9 (90%)	10 (100%)	10 (100%)	87.5 ± 8.8	6 (60%)
I. Medicine	2 (40%)	4 (80%)	4 (80%)	76 ± 26.3	3 (60%)
E. Department	5 (100%)	5 (100%)	5 (100%)	100 ± 0	5 (100%)
**Total**	**65 (76.4%)**	**82 (96.4%)**	**72 (84.7%)**	**84.8 ± 17.8**	**64 (75.2%)**

Table S18: Case 18. Sinus tachycardia with AV block (Mobitz I) and lateral ischemia

SP	ADx [n (%)]	AxC Yes [n (%)]	SDB Yes [n (%)]	CS (Mean ± SD)	AIES [n (%)]
Cardiology	38 (76%)	43 (86%)	33 (66%)	73.4 ± 23.5	40 (80%)
Nurse	11 (73.3%)	8 (53.3%)	7 (46.6%)	72.8 ± 20.6	11 (73.3%)
G. Practitioner	6 (60%)	6 (60%)	8 (80%)	72.5 ± 24.6	6 (60%)
I. Medicine	4 (80%)	3 (60%)	2 (40%)	69.2 ± 39.3	4 (80%)
E. Department	4 (80%)	5 (100%)	5 (100%)	100 ± 0	4 (80%)
**Total**	**61 (71.8%)**	**65 (76.4%)**	**55 (64.7%)**	**74.5 ± 23.9**	**65 (76.4%)**

Table S19: Case 19. Atypical atrial flutter

SP	ADx [n (%)]	AxC Yes [n (%)]	SDB Yes [n (%)]	CS (Mean ± SD)	AIES [n (%)]
Cardiology	32 (64%)	49 (98%)	46 (92%)	86.8 ± 13.4	40 (80%)
Nurse	13 (86.6%)	13 (86.6%)	14 (93.3%)	88 ± 10.5	11 (73.3%)
G. Practitioner	8 (80%)	10 (100%)	9 (90%)	90 ± 11.7	8 (80%)
I. Medicine	5 (100%)	5 (100%)	5 (100%)	97.4 ± 4.2	3 (60%)
E. Department	5 (100%)	5 (100%)	5 (100%)	99 ± 2.2	5 (100%)
**Total**	**63 (74.1%)**	**82 (96.4%)**	**79 (92.9%)**	**88.7 ± 12.3**	**67 (78.8%)**

Table S20: Case 20. Typical atrial flutter

SP	ADx [n (%)]	AxC Yes [n (%)]	SDB Yes [n (%)]	CS (Mean ± SD)	AIES [n (%)]
Cardiology	36 (72%)	50 (100%)	31 (62%)	73.5 ± 23.9	41 (82%)
Nurse	7 (46.7%)	14 (93.3%)	14 (93.3%)	86.5 ± 11.2	10 (66.6%)
G. Practitioner	7 (70%)	9 (90%)	5 (50%)	71 ± 26.4	7 (70%)
I. Medicine	2 (40%)	4 (80%)	5 (100%)	80 ± 16.4	2 (40%)
E. Department	4 (80%)	5 (100%)	5 (100%)	100 ± 0	4 (80%)
**Total**	**56 (65.8%)**	**82 (96.4%)**	**60 (70.5%)**	**77.4 ± 22.5**	**64 (75.2%)**

Table S21: Survey Results: Total (sum) of 20 Cases and by Specialty

SP	YoE (Mean ± SD)	AxC Yes [n (%)]	SDB Yes [n (%)]	CS (Mean ± SD)	AIES [n (%)]
Cardiology	10.0 ± 8.0	981 (98.1%)	802 (80.2%)	81.0 ± 22.2	775 (77.5%)
Nurse	21.6 ± 10.0	260 (86.7%)	223 (74.3%)	79.0 ± 21.2	232 (77.3%)
G. Practitioner	13.3 ± 12.4	192 (96.0%)	155 (77.5%)	78.1 ± 24.8	121 (60.5%)
I. Medicine	6.2 ± 4.1	90 (90.0%)	80 (80.0%)	84.2 ± 23.4	68 (68.0%)
E. Department	22.4 ± 15.0	99 (99.0%)	90 (90%)	91.3 ± 24.2	90 (90%)
**Total**	**13.0 ± 10.4**	**1622 (95.4%)**	**1350 (79.4%)**	**81.1 ± 22.7**	**1286 (75.6%)**

Table S22: Survey Results in Ischemic Cardiomyopathy: Cases 6,7,13,15 and 18

SP	AxC Yes [n (%)]	SDB Yes [n (%)]	CS (Mean ± SD)	AIES [n (%)]
Cardiology	241 (96.4%)	218 (87.2%)	85.4 ± 18.2	193 (77.2%)
Nurse	63 (84.0%)	61 (81.3%)	81.5 ± 19.6	61 (81.3%)
G. Practitioner	45 (90.0%)	42 (84.0%)	79.5 ± 20.9	29 (58.0%)
I. Medicine	23 (92%)	21 (84.0%)	90.0 ± 20.3	17 (68.0%)
E. Department	25 (100.0%)	24 (96.0%)	95.2 ± 20.0	23 (92.0%)
**Total**	**397 (93.4%)**	**366 (86.1%)**	**84.9 ± 19.2**	**323 (76.0%)**

Table S23: Survey Results in Pacemaker Rhythms: Cases 9,10 and 14

SP	AxC Yes [n (%)]	SDB Yes [n (%)]	CS (Mean ± SD)	AIES [n (%)]
Cardiology	147 (98%)	49 (32.7%)	54.4 ± 25.0	145 (96.7%)
Nurse	37 (82.2%)	12 (26.7%)	58.6 ± 24.6	43 (95.6%)
G. Practitioner	29 (96.7%)	9 (30.0%)	50.6 ± 30.9	23 (76.7%)
I. Medicine	13 (86.7%)	5 (33.3%)	63.7 ± 29.3	14 (93.3%)
E. Department	15 (100.0%)	7 (46.7%)	59.2 ± 41.8	14 (93.3%)
**Total**	**241 (94.5%)**	**82 (32.2%)**	**55.5 ± 27.1**	**239 (93.7%)**

Table S24: Survey Results in AV or Bundle Branch Blocks: Cases 1, 3, 4, 6, 8, 11, 16 and 18

SP	AxC Yes [n (%)]	SDB Yes [n (%)]	CS (Mean ± SD)	AIES [n (%)]
Cardiology	387 (96.8%)	349 (87.2%)	83.4 ± 18.9	291 (72.8%)
Nurse	100 (83.3%)	86 (71.7%)	76.5 ± 19.8	90 (75.0%)
G. Practitioner	76 (95.0%)	68 (85.0%)	80.6 ± 21.9	42 (52.5%)
I. Medicine	34 (85.0%)	32 (80.0%)	83.2 ± 25.5	27 (67.5%)
E. Department	39 (97.5%)	39 (97.5%)	96.8 ± 12.0	34 (85.0%)
**Total**	**636 (93.5%)**	**574 (84.4%)**	**82.6 ± 20.0**	**484 (71.2%)**

Table S25: Survey Results in Atrial Arrhythmias: Cases 5, 17, 19 and 20

SP	AxC Yes [n (%)]	SDB Yes [n (%)]	CS (Mean ± SD)	AIES [n (%)]
Cardiology	199 (99.5%)	168 (84.0%)	84.5 ± 18.5	148 (74.0%)
Nurse	53 (88.3%)	55 (91.7%)	88.1 ± 12.2	36 (60.0%)
G. Practitioner	39 (97.5%)	33 (82.5%)	83.8 ± 18.5	26 (65.0%)
I. Medicine	18 (90.0%)	19 (95.0%)	88.1 ± 17.8	9 (45%)
E. Department	20 (100.0%)	20 (100.0%)	99.8 ± 1.1	18 (90.0%)
**Total**	**329 (96.8%)**	**295 (86.8%)**	**86.1 ± 17.3**	**237 (69.7%)**

Signal-processing script for augmented limb lead generation

The following MATLAB function was used to compute the augmented limb leads (aVR, aVL, and aVF) from the standard limb leads (I, II, and III) using the Einthoven–Goldberger relationships.

```
function [D4,D5,D6] = aumentadas(ecg_1,ecg_2,ecg_3)

ecg_4 = -(ecg_1 + ecg_2)/2;
ecg_5 = (ecg_1 - ecg_3)/2;
ecg_6 = (ecg_2 + ecg_3)/2;

D4 = ecg_4;
D5 = ecg_5;
D6 = ecg_6;

tx = linspace(0,30,length(ecg_1));

subplot(3,2,1);
plot(tx,ecg_1/1000);
title('DI','FontSize',14);
xlabel('Time (s)');
ylabel('Amplitude');
grid on;

subplot(3,2,2);
plot(tx,ecg_2/1000);
title('DII','FontSize',14);
xlabel('Time (s)');
ylabel('Amplitude');
grid on;

subplot(3,2,3);
plot(tx,ecg_3/1000);
title('DIII','FontSize',14);
xlabel('Time (s)');
ylabel('Amplitude');
grid on;

subplot(3,2,4);
plot(tx,ecg_4/1000);
title('aVR','FontSize',14);
xlabel('Time (s)');
ylabel('Amplitude');
grid on;

subplot(3,2,5);
plot(tx,ecg_5/1000);
title('aVL','FontSize',14);
xlabel('Time (s)');
ylabel('Amplitude');
grid on;

subplot(3,2,6);
plot(tx,ecg_6/1000);
title('aVF','FontSize',14);
xlabel('Time (s)');
ylabel('Amplitude');
grid on;

end
```
